# A Systems Biology Approach to Understand the Racial Disparities in Colorectal Cancer

**DOI:** 10.1158/2767-9764.CRC-22-0464

**Published:** 2024-01-12

**Authors:** Annabelle Nwaokorie, Walter Kolch, Dirk Fey

**Affiliations:** 1 Systems Biology Ireland, School of Medicine, University College Dublin, Belfield, Dublin, Ireland.; 2 Conway Institute of Biomolecular and Biomedical Research, University College Dublin, Belfield, Dublin, Ireland.

## Abstract

Racial disparities between Black/African Americans (AA) and White patients in colorectal cancer are an ever-growing area of concern. Black/AA show the highest incidence and have the highest mortality among major U.S. racial groups. There is no definite cause other than possible sociodemographic, socioeconomic, education, nutrition, delivery of healthcare, screening, and cultural factors. A primary limitation in this field is the lack of and small sample size of Black/AA studies. Thus, this study aimed to investigate whether differences in gene expression contribute to this ongoing unanswered racial disparity issue. In this study, we examined transcriptomic data of Black/AA and White patient cohorts using a bioinformatic and systems biology approach. We performed a Kaplan–Meier overall survival analysis between both patient cohorts across critical colorectal cancer signal transduction networks (STN), to determine the differences in significant genes across each cohort. Other bioinformatic analyses performed included PROGENy (pathway responsive genes for activity inference), RNA sequencing differential expression using DESeq2, multivariable-adjusted regression, and other associated Kaplan–Meier analyses. These analyses identified novel prognostic genes independent from each cohort, 176 differentially expressed genes, and specific patient cohort STN survival associations. Despite the overarching limitation, the results revealed several novel differences in gene expression between the colorectal cancer Black/AA and White patient cohorts, which allows one to dive deeper into and understand the behavior on a systems level of what could be driving this racial difference across colorectal cancer. Concretely, this information can guide precision medicine approaches tailored specifically for colorectal cancer racial disparities.

**Significance:**

The purpose of this work is to investigate the racial disparities in colorectal cancer between Black/AA and White patient cohorts using a systems biology and bioinformatic approach. Our study investigates the underlying biology of each patient cohort. Concretely, the findings of this study include disparity-associated genes and pathways, which provide a tangible starting point to guide precision medicine approaches tailored specifically for colorectal cancer racial disparities.

## Introduction

Colorectal cancer is the third most common cancer diagnosed and the second leading cause of cancer-related mortality worldwide (1–4). The distribution of colorectal cancer is not even across the worldwide population, there is an evident difference in colorectal cancer incidence and mortality by race and ethnicity. Although colorectal cancer incidence and mortality rates have been declining in recent years, Black/African Americans (AA) show the highest incidence and have the highest mortality among major U.S. racial groups (5–8). During 2014–2018, the overall incidence rates for Black/AA and White patients were 42.6 and 36.1 per 100,000, respectively (6). Thus, the corresponding colorectal cancer incidence rate ratio between Black/AA and Whites were 1.18 (5–8). During 2015–2019, the colorectal cancer mortality rates for Black/AA and Whites were 18.1 and 13.4 per 100,000, respectively (6). Similarly, the corresponding colorectal cancer mortality rate ratio between Black/AA and Whites was 1.35 (5–8). Disparities in colorectal cancer between Black/AA and Whites are an ever-growing area of concern. There is no concrete cause for such disparities other than speculation of the following factors sociodemographic, socioeconomic, screening, education, nutrition, delivery of healthcare, and culture (9–16). Although it is known that the human population shares most of their genetic variation, studies suggest that race may provide valuable information in biomedical contexts, just as other clinical categories such as age, and gender, do (17, 18). One critical study focused on microsatellite instability (MSI) status, investigated the molecular profiles in Black/AA and White derived colon cancers, and concluded that there was no difference in MSI frequencies between both cohorts, suggesting that other factors contribute to the racial disparity (19). Quantifiable differences in patient presentations between Black/AA and White patients, such as the relative predominance of right-side colon neoplasia in Black/AA compared with White patients, imply that molecular influences are present (20). Thus, the genetic contributions to Black/AA and White racial disparities in colorectal cancer are still incompletely understood and have inconclusive findings.

Therefore, we hypothesized that by performing a bioinformatic and systems biology analysis on transcriptomic data of the tumour samples of Black/AA and White patient cohorts, we will reveal novel differences in gene expression, including different genes and signal transduction networks (STN) associated with overall patient survival that help to explain the racial differences.

Systems biology approaches can provide system-level insights into cancer cohorts at an individual patient level (21). Most genetic variations in the molecular nature of colorectal cancer change STNs (22, 23). During tumorigenesis, genetic and epigenetic aberrations of genes combine at the network level to determine the definitive phenotypes (24). As race is a phenotype variation, understanding the disparities that lie on a gene level may be explained at a STN level. Studies have shown that biological differences lie in gene expression patterns in the tumors of Black/AA than in those of Whites (25). Thus, we hypothesize that colorectal cancer STNs likely differ between Black/AA and Whites, as they have been shown to differ in non–small cell lung cancer (25). In addition, molecular alterations in cancer genes and associated STNs are used to advise new treatments for precision medicine in cancer (26). It has been shown that these differences in gene expression amongst Black/AA and Whites could translate to different responses to therapies. Because most targeted therapies today focus on STNs (23, 26–28), to investigate the racial disparities in colorectal cancer at a genetic level the critical colorectal cancer STNs must be focused on. Overall, knowledge of the critical STNs associated with a particular cancer type within a systems biology approach can provide insights into patient-specific differences and an understanding of how certain clinical identifiers such as sex, age, race, cancer stage, and site influence the overall survival of each patient (29).

The aims of this study are 4-fold, (i) identify significant genes across critical colorectal cancer STNs associated with survival in Black/AA and White patient cohorts independently, (ii) identify genes that are differentially expressed by race, between Black/AA and White cohorts from critical colorectal cancer STNs, (iii) assess whether colorectal cancer STN pathway activity is associated with survival, both overall and separately by race, and (iv) investigate what features are significantly contributing to the disparities in the Black/AA and White patient cohorts. We examined transcriptomic data of Black/AA and White patient cohorts, consisting of 64 and 285 patients, respectively. The results revealed several novel differences in gene expression between the colorectal cancer Black/AA and White patient cohorts, thus building a basis for guiding precision medicine approaches tailored specifically for colorectal cancer racial disparities.

## Materials and Methods

### Data Acquisition From the Literature

All data used throughout this study was open-source data and obtained from The Cancer Genome Atlas (TCGA) Colorectal Adenocarcinoma Pan Cancer study (30, 31). The datasets used for this study included Genomic from mRNA sequencing and the associated clinical dataset (30, 31). The datasets used comprised a cohort of 349 patients from the race categories, White (285 patients) and Black/AA (64 patients). All patients selected had mRNA expression available. Preprocessing across the datasets was performed before all analyses were complete. Across all datasets, patients who did not have the associated censoring status or disease-free survival months available were removed to ensure that the same patients were overlapping across all. In total, 333 patients, 61 Black/AA and 272 White patients remained across both datasets (Supplementary Table S1). All datasets analyzed and preprocessed for this study can be found in the Supplementary Figs. S1–S9 and Supplementary Tables S1–S8. Consequently, Supplementary Table S2 represents key metrics and preprocessing steps for all datasets used.

The eight critical colorectal cancer STNs used in this study stemmed from the literature (32–34). The network databases WikiPathways, Kyoto Encyclopedia of Genes and Genomes (KEGG), and gene set enrichment analysis (GSEA), were used to create a list of gene sets for each pathway including WNT, Notch, Apoptosis, PI3K-Akt, Cell Cycle, TP53, MAPK, and TGFβ (35–49). Each gene set consisted of every gene listed for each homo sapiens STN pathway on WikiPathways. These gene sets were then used as input scripts to determine which genes are associated with overall survival in Black/AA and White cohorts. All gene sets are found in the Supplementary Tables S3 and S4.

### Kaplan–Meier Survival Analysis

The Kaplan–Meier survival analysis was simulated three times, the first to find the prognostic genes from mRNA sequencing in the Black/AA cohort, the second prognostic genes from mRNA sequencing in the White cohort, and finally to determine the associations between pathway responsive genes for activity inference (PROGENy) pathway activity scores and overall survival. The optimum cutoff for stratifying the patient populations into low and high groups was identified by scanning the group sizes from 10–90 to 90–10 percent splits, where 10–90 means that 10% of the patients were in the low group and 90% of the patients were in the high group and calculating the *P* value for the overall survival difference between the groups using a log-rank test with Yates’ correction. The corresponding HRs were calculated simultaneously using a Cox proportional hazards regression model using group membership as a single binary covariate. The cutoffs were based on TCGA Pan Cancer datasets: mRNA sequencing (333 patients), 272 patients from the White cohort, and 61 patients from the Black/AA cohort. The inputs for the first two simulations were based on the gene sets for each colorectal cancer STN identified above. Estimating the positive FDR for multiple hypothesis testing was used to obtain the adjusted *P* values for each *P* value using the MATLAB mafdr function and the procedure introduced by Storey, John D (50). The output was a Kaplan–Meier curve for the associated gene expressed, indicating the number of patients in the high expression or low expression group with the corresponding statistical values apparent. The inputs for the final simulation were the PROGENy colorectal cancer pathway activity scores, and the output was 10 Kaplan–Meier curves for each colorectal cancer STN for each patient cohort. The number of patients in the high activity or low activity group was identified with the corresponding statistical values. All statistical computations and Kaplan–Meier analyses were performed in MATLAB [version R2021b (9.11.0. 1769968), The MathWorks, Inc.] using the statistics toolbox and the log-rank [www.mathworks.com/matlabcentral/fileexchange/22317-logrank (accessed on April 7, 2021)] and kmplot [www.mathworks.com/matlabcentral/fileexchange/22293-kmplot (accessed on April 7, 2021)] functions from the MATLAB [version R2021b (9.11.0. 1769968), The MathWorks, Inc.] file exchange. MATLAB, RRID (Research Resource Identifier):SCR_001622.

### Differential Gene Expression Analysis Based on the Negative Binomial Distribution

The RStudio package function DESeq2 was to estimate variance-mean dependence in the mRNA count data from the high-throughput sequencing assay, TCGA, Pan Cancer Atlas and test for differential expression between Black/AA and White patient cohorts based on a model using the negative binomial distribution (30, 31). DESeq2 is a technique for differential analysis of count data. It uses shrinkage estimation for dispersions and fold changes to improve stability and interpretability of estimates. DESeq2 allows a greater quantitative analysis focused on the strength instead of the lacking presence of differential expression (51). DESeq2 is a package installed from Bioconductor as an RStudio package [RStudio Team (2021 version 1.4.1717). RStudio: Integrated Development for R. RStudio, PBC URL https://www.rstudio.com/] (51). From the DESeq2 analysis performed between Black/AA and White cohorts 176 (20%) genes investigated were found to be differentially expressed between the cohorts. A full list of the differentially expressed genes (DEG) is found in the Supplementary Table S5. DESeq2 (version 1.32.0) was used in this study (51). DESeq2, RRID:SCR_000154, RStudio, RRID:SCR_000432, Bioconductor, RRID:SCR_006442.

### PROGENy Analysis

The RStudio package function PROGENy was used to obtain pathway scores from TCGA, Pan Cancer Atlas dataset (30, 31). PROGENy is a machine learning–based tool installed from Bioconductor as an RStudio package [RStudio Team (2021 version 1.4.1717). RStudio: Integrated Development for R. RStudio, PBC, URL https://www.rstudio.com/] (52). A PROGENy analysis was performed on Black/AA and White patient cohorts independently, to determine the association between pathway activity scores and overall patient survival. Despite PROGENy's composition of 14 cancer relevant pathways, specifically for this study, only the colorectal cancer pathways were analyzed in depth analyzed (35–41, 53–55). The available colorectal cancer pathways using PROGENy were PI3K, MAPK, TGFβ, WNT, and p53. The associated PROGENy colorectal cancer pathway activity scores for each patient cohort is found in the Supplementary Tables S6 and S7. PROGENy (version 1.14.0) was used in this study (52).

### Cox Proportional Hazards Model Analysis

The RStudio package function Survival was used to fit a Cox proportional hazards regression model on the TCGA, Pan Cancer Atlas dataset (30, 31). The function coxph stems from the package Survival installed from CRAN as an RStudio package [RStudio Team (2021 version 1.4.1717), URL https://CRAN.R-project.org/package=survival. RStudio: Integrated Development for R. RStudio, PBC URL https://www.rstudio.com/] (42, 43). A Cox proportional hazards regression model was performed on Black/AA and White patient cohorts to determine which clinical feature covariates were associated with patients’ survival and differed by race. A <0.*05 P* value was used as the significance level. The package Survival (version 3.3-1) was used in this study (42, 43).

### Data Availability

The authors confirm that the data supporting the findings of this study are available within the article and its Supplementary Materials and Methods. A list of abbreviations used in this study is found in Supplementary Table S8.

## Results

### Patient Cohort

The data in this study were obtained from TCGA Colorectal Adenocarcinoma Pan Cancer study (TCGA, PanCancer; refs. 30, 31). This dataset comprised a cohort of 594 patients from several race categories including, White (285 patients), Black/AA (64 patients), Asian (12 patients), American Indian or Alaska Native (1 patient), and NA (232 patients). Thus, in line with this study, the race categories analyzed were Black/AA and White, comprising 64 and 285 patients, respectively. An overall total of 349 patients (Supplementary Fig. S1). Although the distribution of Black/AA and White patients is skewed, the small number of Black/AA patients was the greatest number of patients from such a cohort available online through open-source cancer genomic datasets (this limitation is addressed in the Discussion section). In Table 1, all clinical data are distributed homogeneously between Black/AA and White cohorts, and the associated median MSI MANTIS (Microsatellite Analysis for Normal Tumor InStability) scores are 0.32 and 0.35, respectively (45, 56). All patients selected had mRNA expression and clinical data available (Table 1). A detailed list of all clinical data for each patient is available in Supplementary Table S1.

**TABLE 1:** Patient demographics and measured clinical variables. The MANTIS score is a score that predicts the patients MSI status. The higher the MANTIS score the greater is the chance that the patient has the MSI-H status (45, 56). The alternative event frequencies for APC, KRAS, TP53, and TTN are the somatically mutated genes across each cohort

	Overall *n* = 349			Black/AA *N* (%) 64 (18%)			White *N* (%) 285 (82%)		
	Median (IQR)	Minimum	Maximum	Median (IQR)	Minimum	Maximum	Median (IQR)	Minimum	Maximum
Age, years	66 (19)	31	90	61 (18)	31	90	67 (18.25)	31	90
MSI MANTIS score	0.35 (0.04)	0.25	1.37	0.32 (0.04)	0.25	1.20	0.35 (0.04)	0.27	1.37
Overall survival, months	22.34 (24.54)	0	147.9	18.82 (19.94)	0	86.56	23.98 (27.45)	0	147.9
Overall survival status	Living *n* = 253 (73%) Deceased *n* = 80 (23%)			Living *n* = 49 (77%) Deceased *n* = 12 (18%)			Living *n* = 204 (72%) Deceased *n* = 68 (23%)		
Sex	Female *n =* 154 (44%) Male *n =* 179 (51%)			Female *n =* 33 (52%) Male *n =* 28 (44%)			Female *n =* 121 (43%) Male *n =* 151 (52%)		
Tumor stage	Stage I = 50 (14%) Stage II = 109 (31%) Stage III = 108 (30%) Stage IV = 47 (13%)			Stage I = 8 (13%) Stage II = 20 (31%) Stage III = 22 (34%) Stage IV = 11 (17%)			Stage I = 42 (15%) Stage II = 89 (31%) Stage III = 86 (30%) Stage IV = 36 (13%)		
Primary site	Ascending Colon = 42 (12%) Cecum = 72 (21%) Descending Colon = 12 (3%) Hepatic Flexure = 13 (4%) Rectosigmoid Junction = 48 (14%) Rectum = 31 (9%) Sigmoid Colon = 73 (21%) Splenic Flexure = 5 (1%) Transverse Colon = 20 (6%)			Ascending Colon = 9 (14%) Cecum = 21 (33%) Descending Colon = 3 (5%) Hepatic Flexure = 1 (2%) Rectosigmoid Junction = 1 (2%) Rectum = 2 (3%) Sigmoid Colon = 14 (22%) Splenic Flexure = 1 (2%) Transverse Colon = 7 (11%)			Ascending Colon = 33 (12%) Cecum = 51 (18%) Descending Colon = 9 (3%) Hepatic Flexure = 12 (4%) Rectosigmoid Junction = 47 (16%) Rectum = 29 (10%) Sigmoid Colon = 59 (21%) Splenic Flexure = 4 (1%) Transverse Colon = 13 (5%)		
Alteration event frequency									
APC	*n =* 263 (75%)			*n =* 49 (77%)			*n* = 214 (75%)		
KRAS	*n =* 147 (42%)			*n =* 31 (48%)			*n =* 116 (39%)		
TP53	*n =* 222 (64%)			*n =* 42 (66%)			*n =* 180 (63%)		
TTN	*n =* 171 (49%)			*n =* 27 (42%)			*n =* 144 (51%)		

Abbreviation: IQR, interquartile range.

### Determining Overall Survival–associated Genes in Black and White Colorectal Cancer Cohorts

Following the literature, we analyzed nine critical colorectal cancer STNs from several network databases, including WikiPathways, GSEA, and KEGG (57–61). They include WNT, TP53, TGFβ, PI3K-Akt, mTOR, MAPK, Cell Cycle, Apoptosis, and Notch STNs (35–41, 53–55). Features, that is, genes from each pathway were obtained from the listed network databases to determine significant genes in the colorectal cancer Black/AA and White cohorts. Altogether, 1143 features were analyzed across all pathways for both cohorts.

**FIGURE 1:**
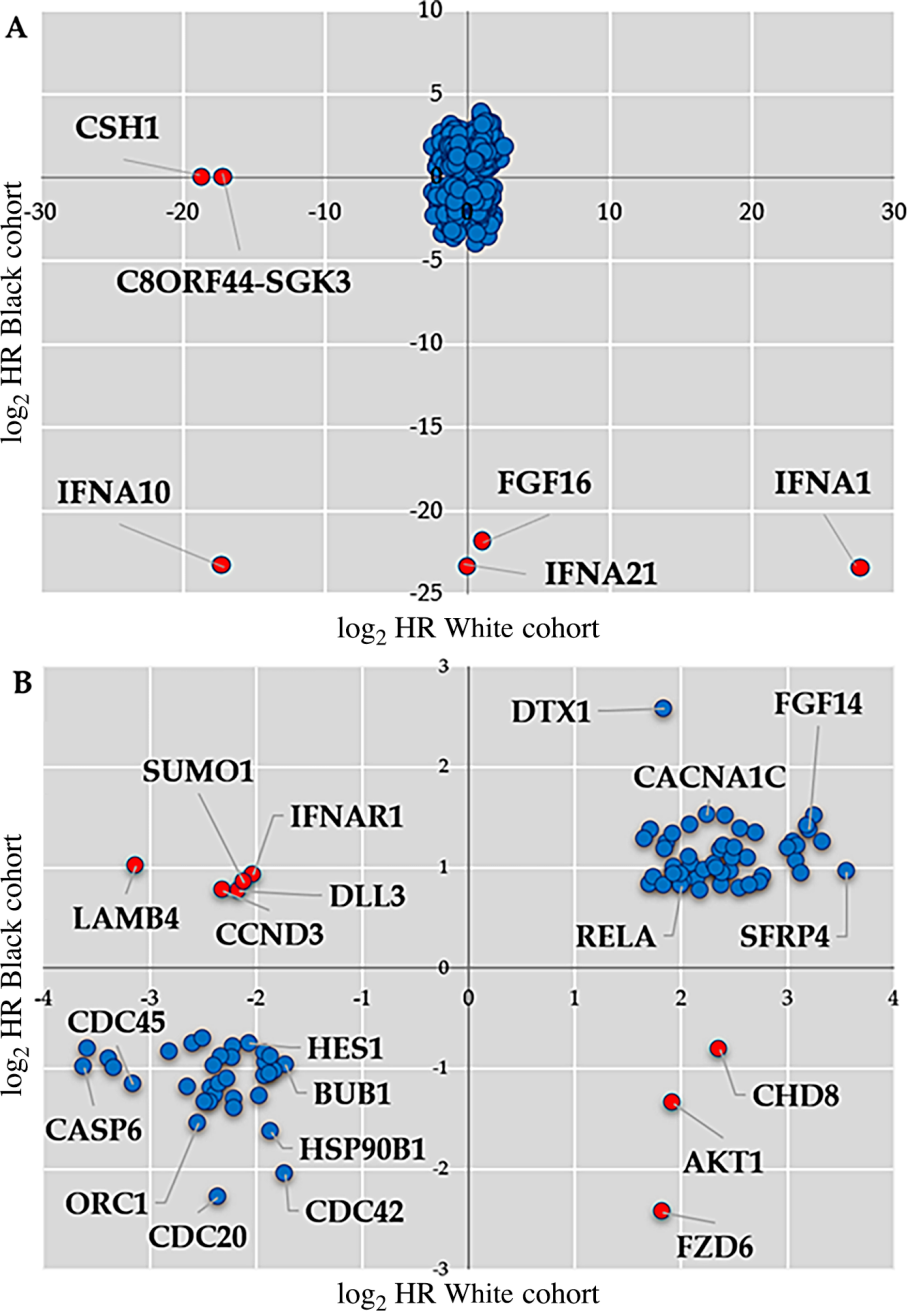
Distribution of the log_2_ HRs from Black/AA and White cohorts. **A,** All genes analyzed in each STN combined. **B,** The subset of genes significant in both cohorts (overlapping significant genes). Blue, genes with concordant survival associations. Red, genes with opposing survival associations between the Black/AA and White cohorts: LAMB4 (HR Black/AA: 2.5976, White: 0.1145), SUMO1 (HR Black/AA: 1.8014, White: 0.2320), IFNAR1 (HR Black/AA: 2.8226, White: 0.2461), DLL3 (HR Black/AA: 1.6974, White: 0.2236), CCND3 (HR Black/AA: 1.6956, White: 0.3233), CHD8 (HR Black/AA: 0.5704, White: 5.1594), AKT1 (HR Black/AA: 0.3929, White: 3.8042), and FZD6 (HR Black/AA: 0.1856, White: 3.5509).

The correlations between patient overall survival and gene expression from a Kaplan–Meier and log-rank test resulted in 811 significant gene associations in both patient cohorts, while MSI status was not associated with overall survival in both cohorts (Supplementary Fig. S2–S4). Across the nine STNs, 218 and 593 significant genes were associated with overall survival for the Black/AA and White cohorts, respectively. The common overlap of significant genes amounts to 110 in total, leaving 701 non-common significant genes between the cohorts. Figure 1 depicts the distribution of the log_2_ HRs of all the genes analyzed in each STN combined and the overlapping common significant genes between Black/AA and White cohorts (Supplementary Fig. S5) While 102 genes had similar survival associations, the sign of the survival associations for eight genes changed between the Black/AA and White cohorts. LAMB4 (laminin subunit beta 4), SUMO1 (small ubiquitin-like modifier 1), IFNAR1 (interferon alpha and beta receptor subunit 1), DLL3 (delta-like canonical Notch ligand 3), CCND3 (Cyclin D3) were associated with increased risk in the Black/AA cohort but decreased risk in the White cohort. On the other hand, CHD8 (chromodomain helicase DNA binding protein 8), AKT1 (AKT serine/threonine kinase 1), and FZD6 (frizzled class receptor 6) were associated with a decreased risk in the Black/AA cohort and increased risk in the White cohort. These results are summarized in Table 2. Interestingly, the top two significant genes were different between the Black/AA and White cohorts for all investigated STNs. The Cell Cycle STN stands out from all other pathways because all top significant genes; YWHAQ (monooxygenase/tryptophan 5-monooxygenase activation protein theta), CCNE1 (cyclin E1), TGFB2 (transforming growth factor beta 2), and ORC1 (origin recognition complex subunit 1) in Black/AA and White cohorts are common between both cohorts. Genes that were found significant between both cohorts are bolded in Table 2. The log_2_ HR distributions of all genes and a list of all significant features within both cohorts are available (Supplementary Table S2; Supplementary Fig. S6).

**TABLE 2:** The top two significant genes associated with overall survival across nine colorectal cancer relevant signaling pathways for both Black/AA and White cohorts from the TCGA, PanCancer Atlas dataset. A Kaplan–Meier estimate, and log-rank test were used to compute the association between patient overall survival and gene expression and report the associated HR, confidence intervals, *P*-value, *P*-adj (*P*-adjusted value), and SE. “Patients” indicates the number of patients for which data were available. “Significant”, indicates the number of significant genes out of the total number of genes for this pathway. The fold change and *P*-value cutoff used were 0.5 and 0.05, respectively. The genes in bold were found to be common top significant genes in both Black/AA and White cohorts in the associated STN (Supplementary Table S3)

	Genes	HR	Confidence intervals	*P*-value	*P*-adj	SE	Patients	Significant
(1) WNT								
Black	WNT7B	7.2703	(1.5885–33.2734)	1.06 × 10^−2^	2.15 × 10^−3^	0.7760	61	23/117
	TCF7L1	5.2885	(1.4183–19.7181)	1.31 × 10^−2^	1.33 × 10^−3^	0.6714		
White	CSNK1A1	0.3271	(0.1840–0.5813)	1.39 × 10^−4^	2.32 × 10^−5^	0.2934	272	67/117
	RAC1	3.4350	(1.7874–6.6012)	2.13 × 10^−4^	1.77 × 10^−5^	0.3333		
(2) TP53								
Black	TP73	0.1998	(0.0585–0.6814)	1.01 × 10^−2^	4.39 × 10^−4^	0.6260	61	5/26
	ATM	5.7337	(1.2398–26.5150)	2.54 × 10^−2^	5.25 × 10^−4^	0.7813		
White	IGFBP3	2.8566	(1.7037–4.7894)	6.87 × 10^−5^	6.03 × 10^−6^	0.2637	272	13/26
	PPM1D	0.4132	(0.2558–0.6673)	3.02 × 10^−4^	1.33 × 10^−5^	0.2446		
(3) TGFβ								
Black	EID2	0.0641	(0.0123–0.3324)	1.07 × 10^−3^	1.60 × 10^−4^	0.8401	61	34/134
	SHC1	6.2661	(1.6939–23.1789)	5.96 × 10^−3^	4.46 × 10^−4^	0.6674		
White	RAC1	3.4350	(1.7874–6.6012)	2.13 × 10^−4^	2.69 × 10^−5^	0.3333	272	68/134
	TERT	3.2047	(1.7110–6.0023)	2.75 × 10^−4^	1.74 × 10^−5^	0.3202		
(4) PI3K Akt								
Black	NTF4	7.3432	(2.1756–24.7844)	1.32 × 10^−3^	7.12 × 10^−2^	0.6206	62	51/338
	BCR	0.0960	(0.0207–0.4445)	2.73 × 10^−3^	7.40 × 10^−2^	0.7823		
White	BRCA1	0.2957	(0.1644–0.5317)	4.70 × 10^−5^	6.91 × 10^−4^	0.2994	271	162/338
	NTRK1	3.7352	(1.9357–7.2076)	8.52 × 10^−5^	8.35 × 10^−4^	0.3353		
(5) mTOR								
Black	EIF4E	0.1732	(0.0464–0.6459)	9.04 × 10^−3^	8.74 × 10^−4^	0.6716	61	2/26
	LAMTOR5	0.2235	(0.0665–0.7503)	1.53 × 10^−2^	7.41 × 10^−4^	0.6179		
White	RRAGB	2.8155	(1.5265–5.1930)	9.19 × 10^−4^	1.81 × 10^−5^	0.3123	272	16/26
	RRAGC	0.4097	(0.2380–0.7053)	1.28 × 10^−3^	1.27 × 10^−5^	0.2771		
(6) MAPK								
Black	HSPA1L	5.9153	(1.7284–20.2437)	4.63 × 10^−3^	8.04 × 10^−2^	0.6277	59	55/253
	CACNA2D4	8.5098	(1.8605–38.9208)	5.77 × 10^−3^	5.01 × 10^−2^	0.7757		
White	TGFB2	3.3496	(1.8537–6.0526)	6.21 × 10^−5^	1.87 × 10^−4^	0.3019	272	130/253
	RASGRP3	3.5062	(1.8914–6.4996)	6.78 × 10^−5^	1.36 × 10^−4^	0.3149		
(7) Cell Cycle								
Black	YWHAQ	0.1790	(0.0525–0.6102)	5.97 × 10^−3^	2.03 × 10^−4^	0.6257	61	20/119
	CCNE1	0.2174	(0.0648–0.7289)	1.34 × 10^−2^	2.28 × 10^−4^	0.6172		
White	TGFB2	3.3496	(1.8537–6.0526)	6.21 × 10^−5^	3.76 × 10^−5^	0.3019	272	68/119
	ORC1	0.3432	(0.2017–0.5840)	8.03 × 10^−5^	2.43 × 10^−5^	0.2712		
(8) Apoptosis								
Black	CASP6	0.0817	(0.0178–0.3747)	1.27 × 10^−3^	1.38 × 10^−3^	0.7773	61	18/84
	DFFA	0.0979	(0.0205–0.4658)	3.50 × 10^−3^	1.91 × 10^−3^	0.7957		
White	IKBKG	3.2997	(2.0251–5.3762)	1.64 × 10^−6^	7.82 × 10^−7^	0.2491	272	42/84
	TRAF1	2.7279	(1.4884–4.9992)	1.17 × 10^−3^	2.78 × 10^−4^	0.3091		
(9) Notch								
Black	NOTCH3	10.0860	(2.1983–46.2749)	2.94 × 10^−3^	1.38 × 10^−3^	0.7773	61	10/46
	JAG2	8.5851	(1.8643–39.5338)	5.79 × 10^−3^	1.35 × 10^−3^	0.7791		
White	DTX1	5.9834	(3.1937–11.2097)	2.33 × 10^−8^	3.83 × 10^−9^	0.3203	272	27/46
	DLL1	2.9814	(1.5902–5.5894)	6.58 × 10^−4^	5.40 × 10^−5^	0.3207		

#### Significant Genes from STN Analysis in the Black/AA Cohort

**FIGURE 2:**
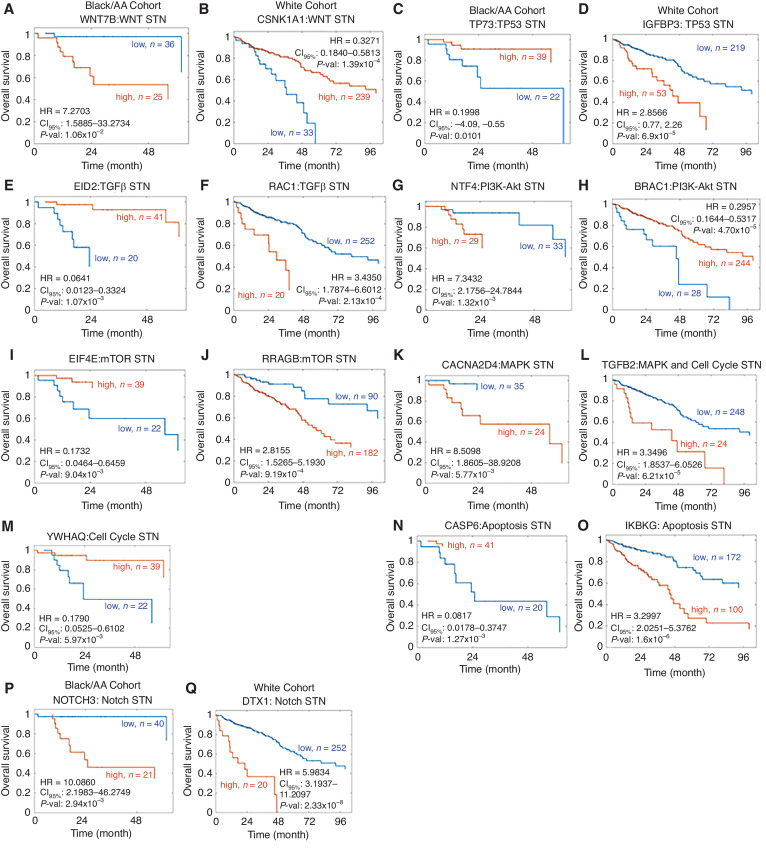
Kaplan–Meier curves of overall survival for the most significant gene of each colorectal cancer STN for the Black/AA and White cohort. **A,** WNT7B for WNT STN for the Black/AA cohort. **B,** CSNK1A1 for WNT STN for the White cohort. **C,** TP73 for TP53 STN for the Black/AA cohort. **D,** IGFBP3 for TP53 STN for the White cohort. **E,** EID2 for TGFβ STN for the Black/AA cohort. **F,** RAC1 for TGFβ STN for the White cohort. **G,** NTF4 for PI3K-Akt STN for the Black/AA cohort. **H,** BRAC1 for PI3K-Akt STN for the White cohort. **I,** EIF4E for mTOR STN for the Black/AA cohort. **J,** RRAGB for mTOR STN for the White cohort. **K,** CACNA2D4 for MAPK STN for the Black/AA cohort. **L,** TGFB2 for MAPK and Cell Cycle STN for the White cohort. **M,** YWHAQ for Cell Cycle STN for the Black/AA cohort. **N,** CASP6 for Apoptosis STN for the Black/AA cohort. **O,** IKBKG for Apoptosis STN for the White cohort. **P,** NOTCH3 for Notch STN for the Black/AA cohort. **Q,** DTX1 for Notch STN for the White cohort. The patients were stratified into two groups according to the expression level of the indicated gene. The optimal cutoff was determined using Kaplan–Meier scanning (see Materials and Methods). The groups are represented as high (orange line), and low (blue line), where *n* indicates the total number of patients in each group. Statistical significance was tested using a log-rank test. HR = hazard ratio. CI_95%_ = 95% confidence interval. *P*-val = *P*-value.

The total number of genes whose RNA expression correlated with overall patient survival in the Black/AA patient cohort was 218 of 1143 genes across the nine colorectal cancer signaling pathways analyzed (Table 2; Fig. 2). Combined for each pathway, around 19% of the pathway genes exhibited an association with overall survival within the Black/AA cohort (fold change and *P*-value cutoffs of 0.5 and 0.05, respectively). A plot of the overall survival Kaplan–Meier curves for the topmost significant genes in the Black/AA cohort is depicted in Fig. 2.

The TGFβ signaling pathway had the largest fraction of significant genes within this cohort, in total 25% of the genes were significant with respect to the number of genes within the pathway. The most significant gene, EID2, exhibited a HR of 0.0641 and a 95% confidence interval between 0.0123 and 0.3324. Thus, a high expression of EID2 resulted in longer patient survival, with a 2-year overall survival of 98% in the EID2-high group (*n* = 41; Fig. 2). TP73 and EIF4E followed the same pattern, where a high expression of these genes signified favorable prognosis. In contrast, a low expression of the genes WNT7B, CACNA2D4, and NOTCH3 correlated with longer overall survival.

#### Significant Genes from STN Analysis in the White Cohort

Across the nine colorectal cancer signaling pathways analyzed within the White cohort, the expression of 593 genes, from a total of 1,143 genes, correlated with overall patient survival (Table 2; Fig. 2). For all pathways, about 51% of the pathway genes exhibited an association with overall survival. A Kaplan–Meier plot of the top significant gene within each pathway for the White cohort is presented in Fig. 2.

**FIGURE 3:**
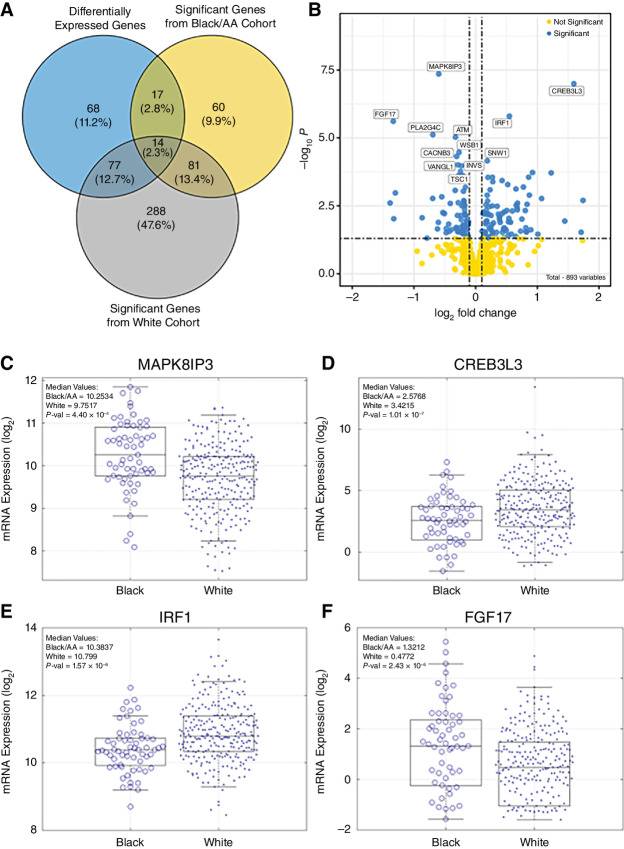
Statistics of DEGs identified by DESeq2. **A,** Venn diagram showing the overlap between the DEGs and the prognostic genes from Black/AA and White cohorts. **B,** Volcano plot showing the DEGs’ *P* values (–log_10_*P*) over the fold change (log_2_). Genes with a *P*-value cutoff <0.05 and absolute log_2_ fold change >0.1 were deemed significant (blue) versus nonsignificant (yellow). Total number of genes analyzed = 893. Comparison of the mRNA distributions for the top four DEGs between the Black/AA and White cohort. **C,** MAPK8IP3 (associated with survival in the White cohort). **D,** CREB3L3 (no association with survival). IRF1 (associated with survival in the Black/AA cohort; **E**), and FGF17 (associated with survival in the White cohort; **F**). On each box, the central line indicates the median, and the bottom and top edges of the box indicate the 25th and 75th percentiles, respectively. *P*-val = *P*-value. All *P* values represented are associated to the DESeq2 analysis.

The mTOR signaling pathway contained the largest fraction of significant genes within this cohort, in total 62% of the genes were significant. The gene IKBKG was the most significant gene in the Apoptosis signaling pathway in the White cohort. IKBKG had a HR of 3.2997 with a 95% confidence interval between 2.0251 and 5.3762 and, *P* value of 1.6 × 10^−6^. High expression of IKBKG was associated with shorter overall patient survival (Fig. 3). In most pathways, approximately 70% of patients in the low expression groups were alive after 2 years, whereas high expressors had significantly reduced overall survival. The only exception was the WNT and PI3K-Akt STN, where high expressions of the most significant genes CSNK1A1 and BRAC1 presented as favourable prognostic markers correlating with longer overall survival.

#### Differential RNA Sequencing Expression Analysis Using DESeq2

The rationale behind performing a differential gene expression analysis in addition to the STN analysis was to determine the differences in genes between Black/AA and White cohorts, that were DEG and or survival-associated genes. The number of unique genes across the nine investigated STNs amounted to 893 genes. Of these, 176 (20%) were found to be differentially expressed between the Black/AA and White cohorts (Supplementary Table S4; Supplementary Fig. S7). For the remaining 717 (80%) genes, there was no statistical evidence of differential expression.

**FIGURE 4:**
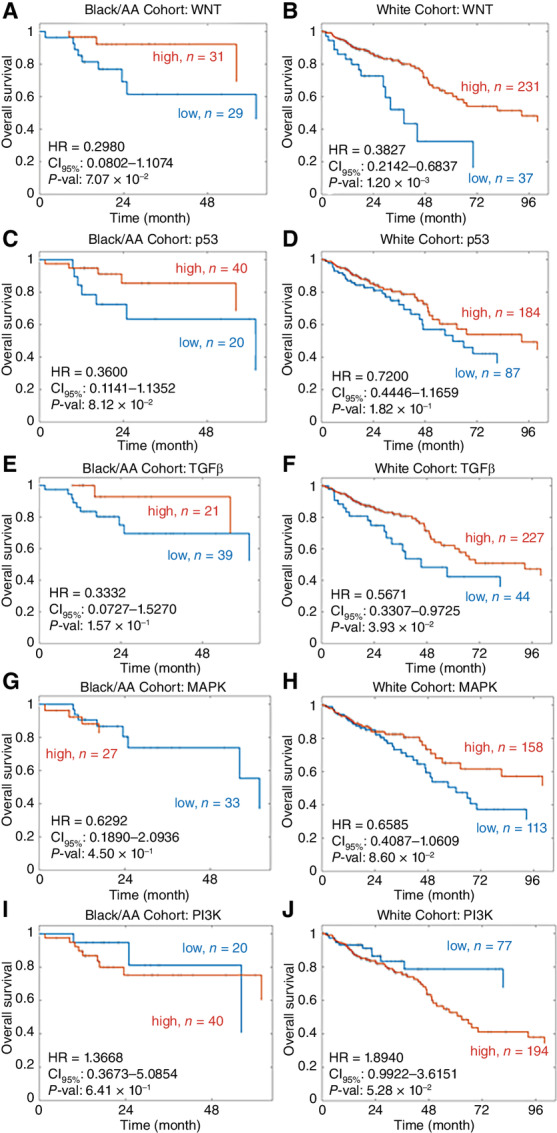
Kaplan–Meier curves of overall survival for the Black/AA and White patient cohorts stratified by pathway activity score. The Kaplan–Meier curves for the signaling pathways WNT for the Black/AA cohort (**A**), WNT for the White cohort (**B**), p53 for the Black/AA cohort (**C**), p53 for the White cohort (**D**), TGFβ for the Black/AA cohort (**E**), TGFβ for the White cohort (**F**), MAPK for the Black/AA cohort (**G**), MAPK for the White cohort (**H**), PI3K-Akt for the Black/AA cohort (**I**), PI3K-Akt for the White cohort (**J**). The groups were based on high (orange line) and low (blue line) pathway activity, where *n* indicates the total number of patients in each group. Statistical significance was tested using a log-rank test. HR = hazard ratio. CI_95%_ = 95% confidence interval. *P*-val = *P*-value.

The list of analyzed genes between both cohorts Black/AA and White and a list of all DEG can be found in the Supplementary Table S4. The intersections between DEG and survival associated genes in the Black/AA and the White cohort are depicted in Fig. 3A. A detailed list of each intersection between DEG and both patient cohorts is shown in Supplementary Fig. S4. 14 genes (2.3% of the 893 unique genes) were both DEG and significantly associated with overall survival in both cohorts, shown in Supplementary Table S4. Conversely, 68 DEG (11.2%) did not exhibit an overall survival association in either cohort (Fig. 4A). A volcano plot analysis (Fig. 3B) identified genes whose differential expression is associated with high (left hand) or low (right hand) risk.

Next, we focused on the top four DEG, and analyzed the corresponding gene expression distributions to understand whether there are any differences in the distributions between the two cohorts. The shape of the distributions between Black/AA and White cohorts followed the same pattern across all top DEG (Fig. 4C–F), this was also mirrored with the survival-associated genes (Supplementary Fig. S6). From these top four DEG, MAPK8IP3 (mitogen-activated protein kinase 8 interacting protein 3), IRF1 (interferon regulatory factor 1), and FGF1 (fibroblast growth factor 17) were also associated with overall survival. MAPK8IP3 found in in the MAPK STN, and FGF17 found in both MAPK and PI3K-Akt STNs were solely associated with survival in the White patient cohort. IRF1, a gene within the Apoptosis STN was associated with survival in the Black/AA cohort independently.

### Analyzing Signaling Pathway Activity Between Black/AA and White Cohorts

After defining a concrete list of the significant genes within the pathways for the Black/AA and White cohorts, we aimed to obtain activity scores for each pathway and patient cohort. The purpose was 3-fold. First, to identify pathway activities that correlate with overall patient survival; second, to relate the gene expression of a pathway to its activity score; and third, to compare the pathway activities and correlations to differential survival between the patient cohorts. For this, we used PROGENy, a method that utilizes publicly available perturbation experiments to identify a common core of genes that respond to activity changes in known signaling pathways and thereby can infer pathway activities from gene expression data obtained under different conditions (52). We focused our analysis on the five colorectal cancer relevant pathways in PROGENy, including PI3K, MAPK, TGFβ, WNT, and p53. The PROGENy activity scores for each colorectal cancer signaling pathway for the Black/AA and White cohorts are shown in the Supplementary Table S5. The results from the activity scores revealed patient-specific differences; different pathways were active in different patients (Fig. 7; Supplementary Fig. S8).

#### Kaplan–Meier Survival Analysis on PROGENy Activity Scores for Black/AA and White Patient Cohorts

To investigate the correlation between the STN activity scores and patient overall survival between Black/AA and White cohorts, a Kaplan–Meier analysis and log-rank test were used. Within the White cohort, out of five pathways, WNT and TGFβ pathway activities were significantly correlated with overall survival (Table 3). In contrast, no significant correlations were observed in the Black/AA cohort. To understand this in more depth, we analyzed the associated Kaplan–Meier curves of the colorectal cancer STNs for both Black/AA and White cohorts.

**TABLE 3:** Survival associations and absolute differences of the pathway activity scores for the Black/AA and White patient cohorts. (**A**) Cox regression was used to analyze the activity score of each pathway. PROGENy scores were analyzed using Kaplan–Meier analysis and the log-rank test to obtain HRs, 95% confidence intervals, *P* values, and SEs. (**B**) The absolute difference between the Black/AA and the White cohorts mean pathway activity scores. *P*-value (two sample *t* test) represents the associated *P*-value from a two-sample *t* test

(A)					
Cohort	Pathways	HR	Confidence intervals	*P*-value	SE
Black Cohort	WNT	0.2980	(0.0802–1.1074)	0.0707	0.6697
	p53	0.3600	(0.1141–1.1352)	0.0812	0.5860
	TGFβ	0.3332	(0.0727–1.5270)	0.1571	0.7767
	MAPK	0.6292	(0.1890–2.0936)	0.4501	0.6134
	PI3K	1.3668	(0.3673–5.0845)	0.6411	0.6703
White Cohort	WNT	0.3827	(0.2142–0.6837)	0.0012	0.2960
	TGFβ	0.5671	(0.3307–0.9725)	0.0393	0.2752
	PI3K	1.8940	(0.9922–3.6151)	0.0528	0.3298
	MAPK	0.6585	(0.4087–1.0609)	0.0860	0.2433
	p53	0.7200	(0.4446–1.1659)	0.1817	0.2459
**(B)**					
		**Activity scores**			
	**Pathway**	**Black/AA**	**White**	**|Difference|**	**P(T≤t) two-tail**
	WNT	0.1253	0.1168	0.0085	1
	MAPK	0.1033	−0.0416	0.1449	1
	PI3K	0.0372	−0.0224	0.0596	1
	TGFβ	0.0793	0.0649	0.0144	1
	P53	0.0153	0.0392	0.0239	1

The Black/AA cohort overall survival Kaplan–Meier curves for the colorectal cancer STNs WNT, p53, TGFβ, MAPK, and PI3K-Akt are shown in Fig. 4A–E. None of the STN activity scores were associated with patient survival. However, trends were visible. The WNT, p53, MAPK, and TGFβ STNs exhibited a nonsignificant association of high activity score with longer overall survival. The correlation of high WNT activity and longer overall survival was found to be counterintuitive. In addition, the results for the TGFβ STN are also counterintuitive. For the TGFβ signaling pathway, the HR was 0.3332 with a 95% confidence interval between 0.0727 and 1.5270 and a *P* value of 0.1571. The high group characterized by high TGFβ activity consisted of 21 Black/AA patients and the low group of 39 Black/AA patients. For the White cohort, the overall survival Kaplan–Meier curves for the colorectal cancer STNs WNT, p53, TGFβ, MAPK, and PI3K-Akt are shown in Fig. 4F–J. Three of these STNs, WNT, TGFβ, and PI3K-Akt activity scores were associated with overall patient survival. High activity scores of WNT and TGFβ STNs were associated with longer overall survival. As mentioned above this is not what one would expect. High activity scores of the PI3K-Akt STN were associated with shorter overall survival, which is expected from the literature. The results for the PI3K-Akt signaling pathway for the White patient cohort include a HR of 1.8940 with a 95% confidence interval between 0.9922 and 3.6151 and a *P* value of 0.0528. A high activation of this pathway was seen in 194 patients and a low activation in 77 patients.

Comparing both Black/AA and White cohorts, a potentially interesting racial difference emerges between the associations of pathway activity scores and patient overall survival. To build confidence in these pathway activity scores and to gain a deeper understanding of which factors are associated with patient survival, the next step was to perform a multivariable-adjusted regression survival analysis using Cox proportional hazards model.

The patients are categorized into APC wildtype and APC mutant groups for both Black/AA and White patients (Supplementary Fig. S9). All distributions of each group follow a similar trend; most patients in each cohort are APC mutant rather than wildtype, proving dependability in the WNT activity scores. A Cox proportional hazards model was used to evaluate simultaneously the effects of the WNT activity scores, stage, and race on overall patient survival. The WNT STN was investigated because WNT activity was the most significant STN in the White cohort and marginally significant in the Black/AA cohort. Because APC mutations can activate the WNT STN, we should be able to see an association between the patients APC mutation status and WNT activity score (44). The HR and the 95% confidence intervals for each covariate included in the Cox proportional hazards model are presented the Supplementary Fig. S9. The WNT activity scores and stage provide significance and contribute to patient survival. The associated *P* values are 0.023 and 0.019, respectively.

### What Features are Significantly Contributing to the Disparities in the Black/AA and White Patient Cohorts Through a Multivariable-adjusted Regression Cox Analysis

#### Are the Clinical Features, Sex, Site, Stage, and Age, Contributing to the Racial Disparities Found Between Colorectal Cancer Black/AA and White Cohorts?

We then sought to analyze what available clinical features, if any, are contributing to the differences in the STN activity scores between patients. The results should allow us to determine whether the racial disparities in colorectal cancer are led by common clinical factors or by confounding factors, including, quality of care, screening programmes, insurance, environmental factors, and socioeconomic status as mentioned in many studies (9–12). The clinical features analyzed include sex, stage, race, tumour location, and age. To investigate whether there were location-specific differences in the colorectal cancer patient cohorts, the clinical factor location was aggregated into three sections: right; the ascending colon: cecum, left; the descending colon: rectum and sigmoid, and other; the transverse colon. Similarly, stage was divided into three sections, stage I–III, stage IV, or metastasis, and other. The goal behind segregating by stage was to determine stage-specific differences in STN activity scores. In addition, age was categorized by premenopausal and postmenopausal, <50 and >50 years, respectively. Our clustering results showed no clear correlation between the clinical features (age, stage, race, site, and sex) and the STN activity scores. This suggests that the clinical features are not related to race-specific pathway activity scores.

#### Multivariable-adjusted Regression Analysis Between Black/AA and White Patient Cohorts

**FIGURE 5:**
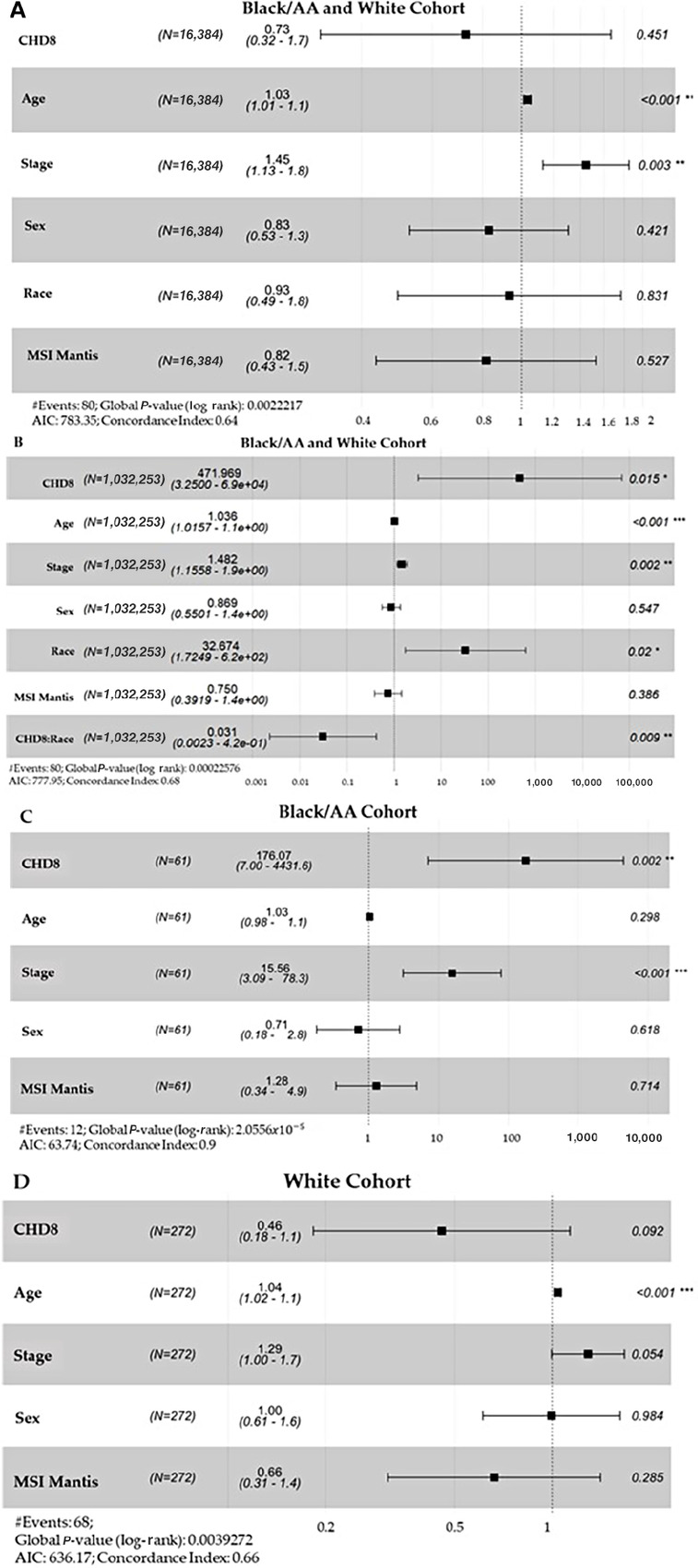
Multivariable-adjusted regression survival analysis of indicated variables. Forest plots of the Cox proportional hazard ratio analysis of the Black/AA and White patient cohorts. Clinical identifiers age, stage, sex, race, MSI MANTIS, and CHD8 (**A**) of the WNT STN was a gene significantly associated with survival in both cohorts, see Fig. 1, (**B**) the same covariates as A, with the inclusion of the interaction effect covariate, CHD8:Race (**C**) and (**D**) CHD8, age, stage, sex, and MSI MANTIS for the Black/AA and the White, respectively. The first column represents the clinical identifier of interest. The number of events = 80 and the number of patients *n* = 333, 62 Black patients and 271 White patients. *N*, the second column is the number of observations deleted because of missingness. The third column represents the HR and the 95% confidence intervals of the HR for each covariate included in the Cox proportional hazards model. The HR estimates are depicted by box symbols with confidence bands and parenthetical values representing 95% confidence intervals. The last column indicates the associated *P* value, which is represented by the Wald test of significance. Magnitude of significance is denoted with asterisks (*). The significance codes include: 0 “***” 0.001 “**” 0.01 “*” 0.05 “.” 0.1 “ ” 1. AIC = Akaike information criterion.

The final part of this study analyzed three Cox proportional hazards models between Black/AA and White cohorts. The aim was to decipher what clinical features, if any, are associated with patients’ survival and differ by race (Fig. 5). The covariates of interest are critical clinical features that might contribute to patient survival. These features include age, stage, sex, race, and the MSI MANTIS score [This score that predicts the patients MSI status. The higher the MANTIS score, the greater chance the patient is to have the microsatellite instability-high (MSI-H) status (45)]. For the genetic feature, we focused on CHD8, because this gene was associated with patient survival in both cohorts from the WNT signaling pathway. In addition, this gene was found to have opposed survival associations in both cohorts (Fig. 1). The associated HR values for CHD8 in the Black/AA cohort were 0.5704 and 5.1594 in the White cohort.

When race was adjusted as a covariate (Fig. 5A), CHD8 did not provide significance and contribute to patient survival between the Black/AA and White patient cohorts. The associated *P* value was 0.451. Thus, Fig. 5B shows the importance of adding an interaction effect between CHD8 and race, as the associated *P* value of the interaction effect was 0.009. The coefficient of the interaction effect is negative unlike the positive coefficient of CHD8, consequently, both covariates clearly display opposite outcomes indicating the effect is opposite in Black/AA and White cohorts. Overall, this is finding confirmed in Fig. 5C and D, where we analyzed the two cohorts separately. CHD8 was significant in the Black/AA cohort, with a large magnitude of significance with a *P* value of 0.002. For the White cohort, a *P* value of 0.092 was found. Overall, the current results support the importance of evaluating associations between gene expression and survival separately by race by (i) including an interaction effect to capture the effect of race, or (ii) stratifying by race.

## Discussion

Our analysis workflow employed numerous Kaplan–Meier survival scans, a differential mRNA expression analysis, a pathway activity score analysis, and Cox proportional hazards models to investigate features differentially contributing to Black/AA and White patients’ survival. The results can serve as starting point for directing precision medicine approaches tailored for colorectal cancer racial disparities. Conversely, the literature to date has insufficient explanations for the increased mortality and incidence rates for Black/AA patients in comparison to White patients, other than the expected factors of sociodemographic, socioeconomic, education, screening, nutrition, delivery of healthcare, and culture (9–16). Exploring the possibility of differential gene expression contributing to the differences in Black/AA patient survival, our work identifies novel survival-associated genes, DEGs, and colorectal cancer STN activity associations with survival while also accounting for clinical covariates. Overall, our results provide novel potential factors that may influence the high mortality of Black/AA patients. The identified gene and pathway differences could be a starting point for exploring racial disparities between Black/AA and White patients with colorectal cancer.

### Overall Survival Analysis

This study determined genes significantly associated with overall survival in colorectal cancer across the major eight colorectal cancer signaling pathways, WNT, PI3K-Akt, TP53, MAPK, Apoptosis, Notch, Cell Cycle, and TGFβ. LAMB4, IFNAR1, DLL3, and CCND3 were associated with increased risk in the Black/AA cohort and decreased risk in the White cohort, with associated *P* values for interaction with race of 0.423, 0.840, 0.337, and 0.454, respectively. While CHD8, AKT1, and FZD6, were associated with decreased risk in the Black/AA cohort and increased risk in the White cohort, with associated *P* values for interaction with race of 0.009, 0.712, and 0.618, respectively. These results suggest that the meaning of these eight genes, that is, how they must be interpreted, changes between the Black/AA and White cohorts. We hypothesize that the reason for this seen in recent advances by, Mitchell and colleagues, is associated with biological differences in gene expression patterns in the tumors of Black/AA than in those of Whites in certain colorectal cancer STNs (25). These expression patterns constitute different contexts, which influence the function of some colorectal cancer STNs to differ between Black/AA and Whites as shown in non–small cell lung cancer (25). In addition, it has been shown that these differences in gene expression among Black/AA and Whites could translate to different responses to therapies (23, 26–28). Overall, this result highlights that research needs to focus on the colorectal cancer STNs that may play a role in the destructive mortality and incidence of colorectal cancer in Black/AA patients. Apart from genetics, these associations might be influenced by other factors, including environmental, socioeconomic status, education, nutrition, screening, delivery of healthcare, and culture (9–16).

For the Black/AA cohort, the number of genes associated with survival was only 19% compared with 51% in the White cohort. This discrepancy could be due to the higher patient numbers in the White cohort which results in a higher statistical power and more survival-associated genes. The TGFβ signaling pathway had the largest fraction (25%) of significant genes within this cohort. The gene with the highest association with survival was EID2 in this STN. Previous studies demonstrated that a high expression of EID2 in colorectal cancer acts as a favourable prognostic marker (46). Our study solidifies this, as shown in the Kaplan–Meier curve in Fig. 2, in which a high expression of EID2 was associated with a higher overall patient survival. The same pattern was followed by TP73 and EIF4E, the topmost genes associated with survival in the TP53 and mTOR STNs, respectively, where a high expression implies a favorable prognostic marker (Fig. 2). Kotulak and colleagues support this finding as they suggest that TP73 may play a role as a tumor suppressor in colorectal cancer progression (47). Conversely, the potent oncogene, EIF4E, overexpression accounts for approximately 30% of cancer cases (48, 49). It plays a critical role in mRNA recruitment (62). EIF4E phosphorylation in response to extracellular stimulation leads to uncontrolled translation and proliferation and inhibits apoptosis (63). Studies show when it is overexpressed it results in more frequent liver metastasis, suggesting the prognostic effect of EIF4E on colorectal liver metastasis (64). Together, these results identify novel genes associated with Black/AA patient survival.

For the White cohort, the largest fraction (62%) of significant genes associated with patient survival came from the mTOR STN, which is different from the Black/AA cohort. The mTOR STN plays a vital role in the regulation of cell survival, metabolism, growth, and protein synthesis, it has emerged as an effective target for colorectal cancer therapy (65, 66). RRAGB expression was most strongly associated with survival in the mTOR STN. This is consistent with the literature where high expression of RRAGB predicted poor overall survival (67). Xiao and colleagues showed that RRAGB expression was significantly associated with MSI, tumor mutational burden (TMB) and immunity. Their results uncovered that RRAGB could be a prognostic biomarker for colon adenocarcinoma in terms of overall survival that is related to MSI, TMB, and immunity (67, 68). Our study confirms these findings, as shown in the Kaplan–Meier curve in Fig. 2, in which a high expression of RRAGB was associated with shorter overall patient survival. Intriguingly, IKBKG was most associated with patient overall survival in the Apoptosis STN within the White cohort. IKBKG follows a similar trend to RRAGB as a high expression correlates with shorter overall patient survival (Fig. 2). Interestingly, IKBKG inhibition suppresses the proliferation of colorectal cancer cells *in vitro* (69). IKBKG encodes the regulatory subunit of the inhibitor of kappaB kinase (IKK) complex, this activates NFκB resulting in activation of genes involved in inflammation, immunity, cell survival, and other STN (70). The NFκB pathway is known as a key regulator of colorectal cancer cell proliferation, apoptosis, angiogenesis, inflammation, metastasis, and drug resistance. In addition, an overactivation of this pathway is a characteristic of colorectal cancer (71). Studies show that anti-NFκB therapy should be considered as a therapeutic target as it may salvage many instances of colorectal cancer (72).

Finally, the results for the overall survival analysis identified several survival-associated genes common and exclusive between Black/AA and White colorectal cancer patient cohorts that have not yet been accounted for in the literature. These novel features can be the starting point into the investigation of the possible factors contributing to colorectal cancer patient survival. To date, there have not been survival-associated gene sets based on the clinical attribute race. Presenting these cohort-specific survival-associated genes can influence precision medicine approaches tailored for colorectal cancer racial disparities. For example, if a gene is prognostic in one cohort but not the other, it might be a good drug target in this cohort, but not the other.

### A DEG Analysis

Several studies in the literature resulted in lists of DEGs which tend to be inconsistent with each other, suggesting that there are some false positives and false negatives (73–75). Investigating the intersections between DEGs and genes associated with overall survival may be a way to limit false positives and negatives and provide a reduced set of more meaningful genes that can be validated further in the future. From the top DEGs, it is interesting to note that some of these genes are associated with overall survival in patient cohorts. MAPK8IP3 found in the MAPK STN, and FGF17 found in both MAPK and PI3K-Akt STNs were solely associated with survival in the White patient cohort. Whereas IRF1 a gene within the Apoptosis STN was associated with survival in only the Black/AA cohort independently. This finding may indicate that certain genes importance in one cohort over another.

### A PROGENy Analysis

The next part of this study consisted of a pathway activity score analysis. We chose to do this PROGENy pathway analysis and not a classical GSEA. Unlike GSEA, PROGENy predicts specific pathway activity scores for each individual patient, thus, allowing us to identify the patterns and behaviors of the activity scores on a personal level (52). The advantage is that the patient-specific scores allow us to reveal differences between any clinical variables of interest (age, stage, race, site, and sex). Interestingly, the PROGENy analysis did not reveal any linked patterns between the clinical features and the STN activity scores. No pronounced clusters were identified that related clinical features to specific pathway activity scores.

Thus, we asked whether the pathway activity scores had any associations with overall patient survival. No associations between the STN activity scores and overall survival crossed our chosen significance threshold (*P* < 0.05) in the Black/AA cohort (Supplementary Table S3; Supplementary Fig. S8). One reason for this could be the low number of patients (*n* = 61) in the Black/AA cohort. The lack of publicly available omics data for Black/AA patients is a major problem when trying to study racial disparities (76). In total, 61 Black/AA patients were analyzed in comparison to 272 White patients, this dramatic contrast will introduce poor statistical power.

In contrast, for the White cohort, three STNs, WNT, TGFβ, and PI3K-Akt activity scores were associated with overall patient survival (Supplementary Table S3; Supplementary Fig. S8). The association of high activity scores of the PI3K-Akt STN with shorter overall survival was expected from the literature. The PI3K-Akt STN has an oncogenic role in the introduction and development of colorectal cancer; it is expected that a high STN activation would result in a shorter patient overall survival (77–79). WNT and TGFβ STNs are associated with longer overall survival, counterintuitive considering that the activation of the WNT pathway increases the levels of β-catenin, causing it to translocate into the nucleus and express WNT target genes that drive cell proliferation (29, 80). More than 90% of colorectal cancers have activating somatic mutations in the WNT pathway (APC loss or β-catenin mutation), thus the WNT pathway activation is considered as a prerequisite for colorectal cancer pathogenesis (29, 80–86). Overall, WNT pathway activation is typically associated with shorter patient survival (81–86). Although TGFβ can have tumor-suppressive functions, acting as a potent inhibitor of normal colonic epithelial cells, it can also have tumor-promoting functions promoting the survival, invasion, and metastasis of colorectal cancer cells, and is known as a tumor promotor in the last stages of colorectal cancer through its immunosuppressive function (77, 87–89). The reason for these discrepancies might be because of the treatment each patient received, as chemotherapy performs better for proliferative cells. Another possibility could have been that the WNT activity scores were not reliable. However, APC-mutated patients exhibited increased WNT activity scores (Supplementary Fig. S9), just as one would expect considering that APC mutations activate the WNT pathway (29, 80–86).

With the development and implementation of precision medicine approaches still being limited among Black/AA patients, one would expect a rise in more diverse omics data among minority populations. Precision medicine is predicted to transform the clinical practice of medicine, by using molecular biomarkers to assess patients’ risk, prognosis, and therapeutic response more accurately (90). However, relying on biomarkers that do not represent a diverse population presents challenges for diagnosing and treating underrepresented populations. Consequently, our study, although small, has revealed novel genetic features that could guide the direction of precision medicine approaches toward accounting for colorectal cancer racial disparities.

### Limitation

A primary limitation of this study is the lack of and relatively small sample size of the Black/AA cohort (64 patients). Table 1 demonstrates several statistical analyses performed to alleviate false discovery, in addition, it shows that the distribution of the clinical data between Black/AA and White patient cohorts is homogeneous. Despite this limitation, we believe the results of this study indicate an extensive amount of value on where to start when analyzing racial disparities. In addition, it is possible that some of the genes associated with survival among White individuals, but not Black/AA individuals, are due to the smaller sample size for the Black/AA cohort. Future studies will reassess the results when another larger dataset becomes available.

### Summary

In summary, we identified novel prognostic genes independent from the Black/AA and White patient cohorts, 176 DEG, and specific patient cohort STN survival associations. The findings display several differences in gene expression between the colorectal cancer Black/AA and White patient cohorts, which aid one to dive deeper into and understand the behavior on a systems level of what could be driving this racial difference across colorectal cancer. Concretely, this information can guide precision medicine approaches tailored specifically for colorectal cancer racial disparities.

## Supplementary Material

Supplementary Table S1 This file is the entire list of clinical data for both patient cohorts used in the study.

Supplementary Table S2 This file contains an overview of the datasets used and the analyses performed.

Supplementary Table S3 This file contains the results to the survival analysis performed across each patient cohort for each signal transduction network.

Supplementary Table S4 This file contains a list of the common top significant survival associated genes in both Black/AA and White cohorts.

Supplementary Table S5 This file contains the results to the differential gene expression analysis, and shows the list of all genes analysed.

Supplementary Table S6 This file contains the PROGENy Activity Score Results from both cohorts for each signalling pathway analysed.

Supplementary Table S7 This file contains the t-tests performed on the PROGENy activity scores.

Supplementary Table S8 This file is a list of abbreviations found in the manuscript

Supplementary Figure S1 Supplementary Figure S1 shows the distributions of the number of patients in each race categories from the TCGA PanCancer Atlas dataset

Supplementary Figure S2 Supplementary Figure S2 shows the MSI MANTIS Score for each patient cohort Black/AA and White

Supplementary Figure S3 Supplementary Figure S3 shows the MSI MANTIS Score distribution for each patient cohort Black/AA and White

Supplementary Figure S4 Supplementary Figure S4 shows the probability of overall survival for the MSI MANTIS Scores for each patient cohort

Supplementary Figure S5 Supplementary Figure S5 shows the distribution of the log2 Hazard Ratios from Black/AA and White cohorts

Supplementary Figure S6 Supplementary Figure S6 shows the mRNA distributions for the most significant gene of each CRC STN found in the Black/AA and White patient cohorts

Supplementary Figure S7 Supplementary Figure S7 shows a heatmap of the differentially expressed genes between the Black/AA and White cohorts

Supplementary Figure S8 Supplementary Figure S8 shows the PROGENy pathway activity scores from the mRNA expressions for Black/AA and White patient cohorts

Supplementary Figure S9 Supplementary Figure S9 shows the overview of the effects of WNT pathway activity scores and APC alternative event frequency between Black/AA and White patient cohorts
