# Supplementary material for: A Systems Biology Approach to Understand the Racial Disparities in Colorectal Cancer
Source: Cancer Res Commun. 2024 Jan 12;4(1):103–17. doi: 10.1158/2767-9764.CRC-22-0464 (PMC10785768; doi:10.1158/2767-9764.CRC-22-0464)
Supplement: Supplementary Figure S1 — shows the distributions of the number of patients in each race categories from the TCGA PanCancer Atlas dataset [file crc-22-0464-s09.docx]

Supplementary Figure S1

***
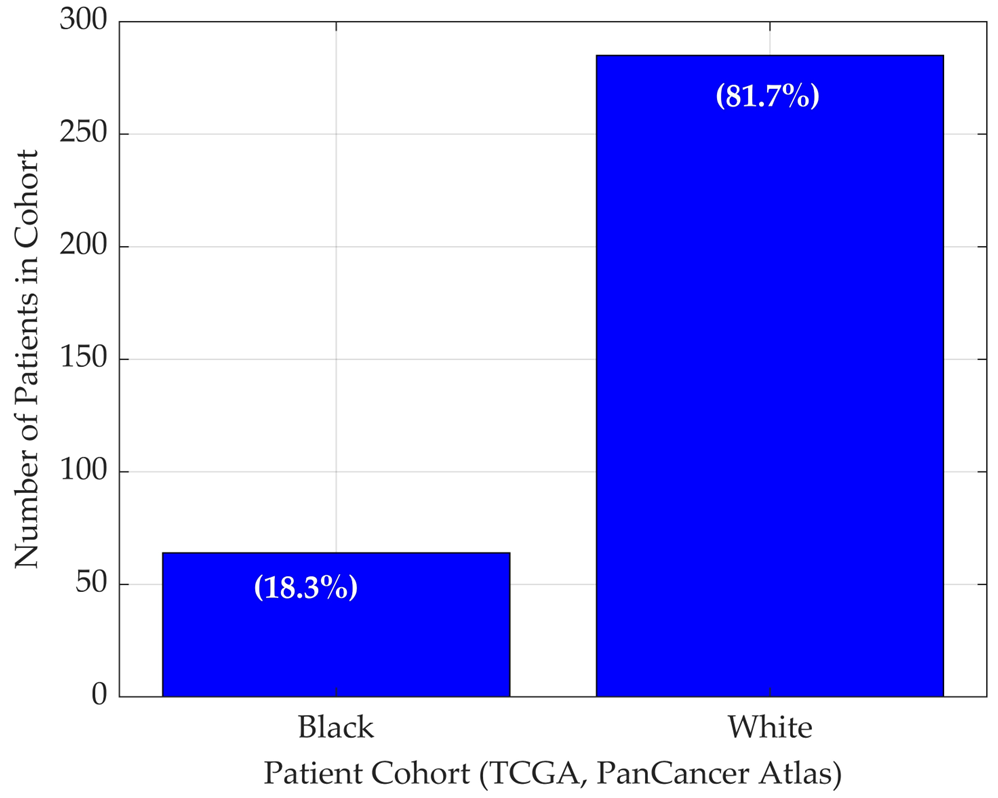
***

**Figure S1. Distributions of the number of patients in each race categories from the TCGA PanCancer Atlas dataset.** Race categories of interest; Black/AA and White. In total the Black/AA and White cohorts were comprised of 64 and 285 patients, respectively. An overall total of 349 patients.
