# Supplementary material for: A Systems Biology Approach to Understand the Racial Disparities in Colorectal Cancer
Source: Cancer Res Commun. 2024 Jan 12;4(1):103–17. doi: 10.1158/2767-9764.CRC-22-0464 (PMC10785768; doi:10.1158/2767-9764.CRC-22-0464)
Supplement: Supplementary Figure S2 — shows the MSI MANTIS Score for each patient cohort Black/AA and White [file crc-22-0464-s10.docx]

Supplementary Figure S2

**
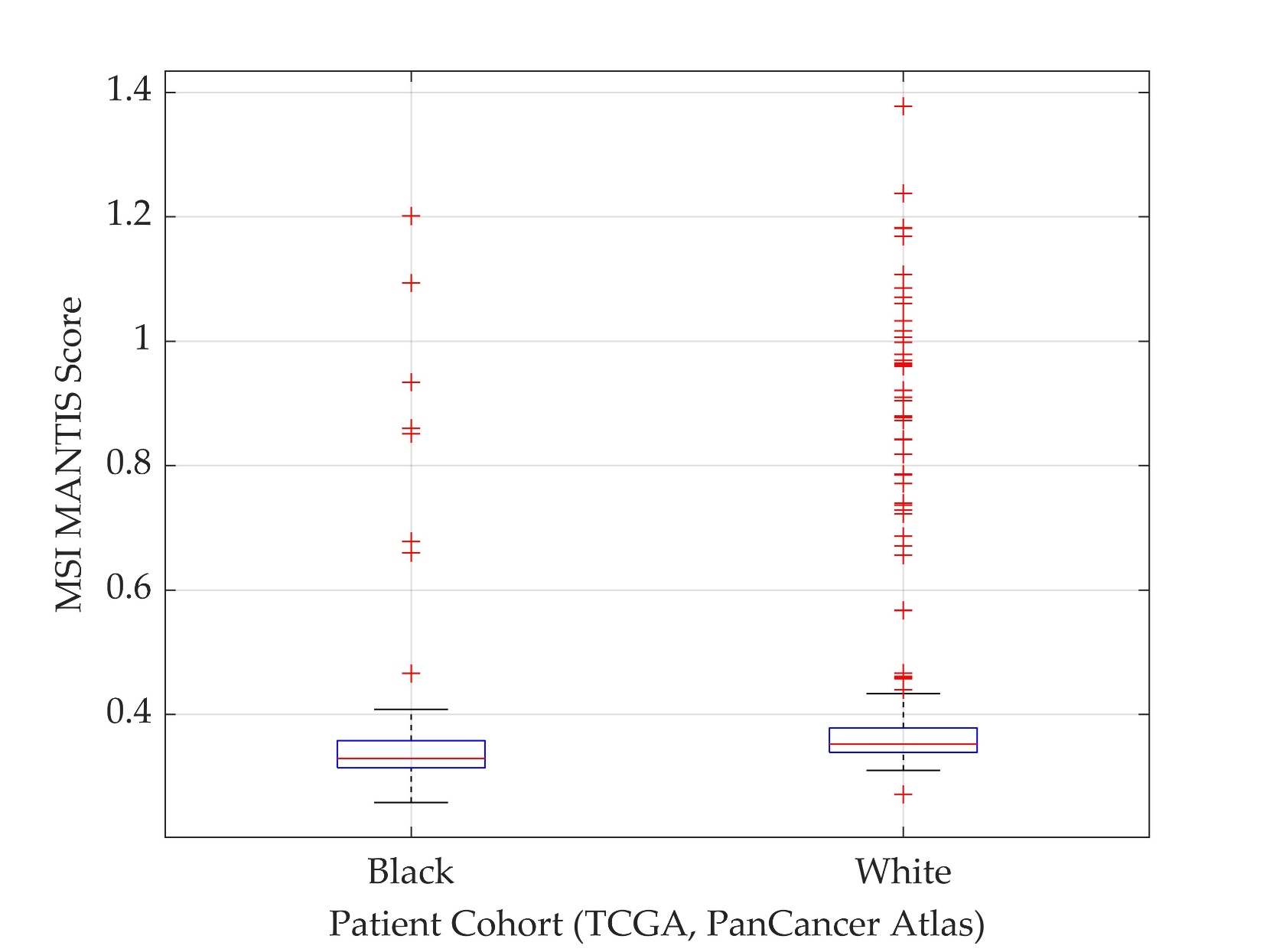
**

**Figure S2. MSI MANTIS Score for each patient cohort Black/AA and White.** The suggested thresholds are MSI: >0.6, Indeterminate: 0.4 - 0.6 and MSS: <0.4. The clinical attribute MSI MANTIS Score was determined to be statistically significant from performing a Wilcoxon test when comparing the two cohorts. The associated *p*-value 9.63${x10}^{-7}$and *q*-value 9.82${x10}^{-6}.$
