# Supplementary material for: A Systems Biology Approach to Understand the Racial Disparities in Colorectal Cancer
Source: Cancer Res Commun. 2024 Jan 12;4(1):103–17. doi: 10.1158/2767-9764.CRC-22-0464 (PMC10785768; doi:10.1158/2767-9764.CRC-22-0464)
Supplement: Supplementary Figure S3 — shows the MSI MANTIS Score distribution for each patient cohort Black/AA and White [file crc-22-0464-s11.docx]

Supplementary Figure S3

**
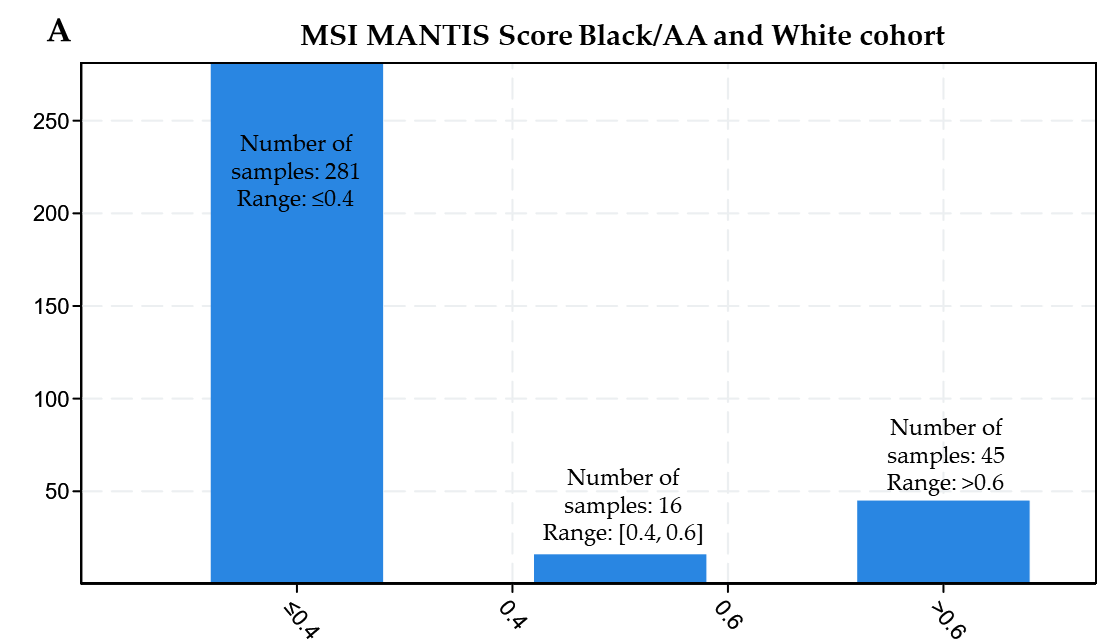

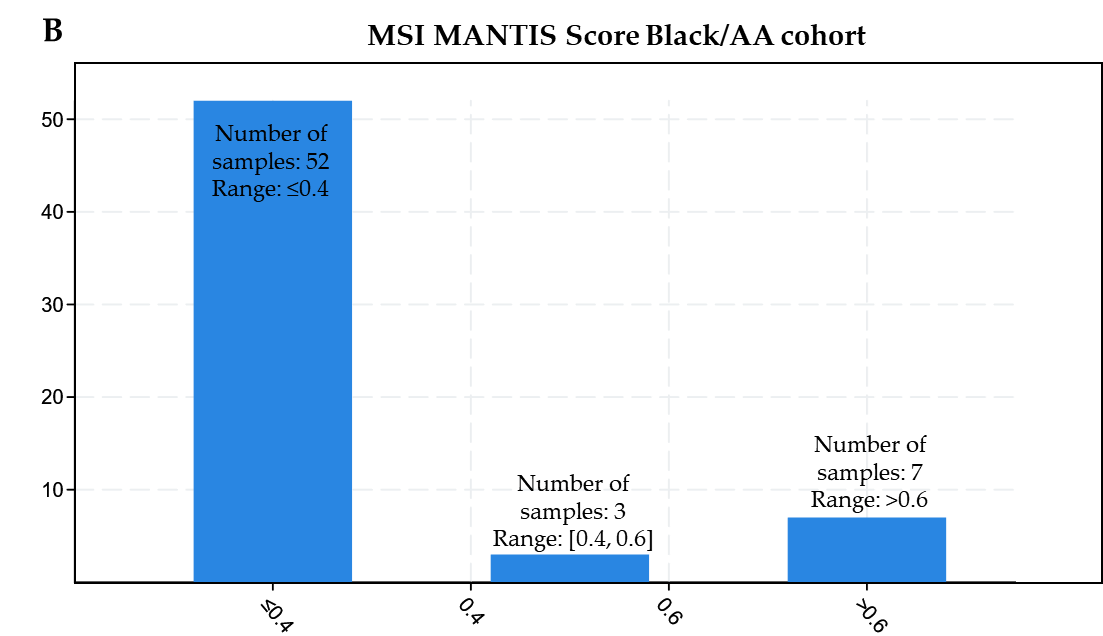

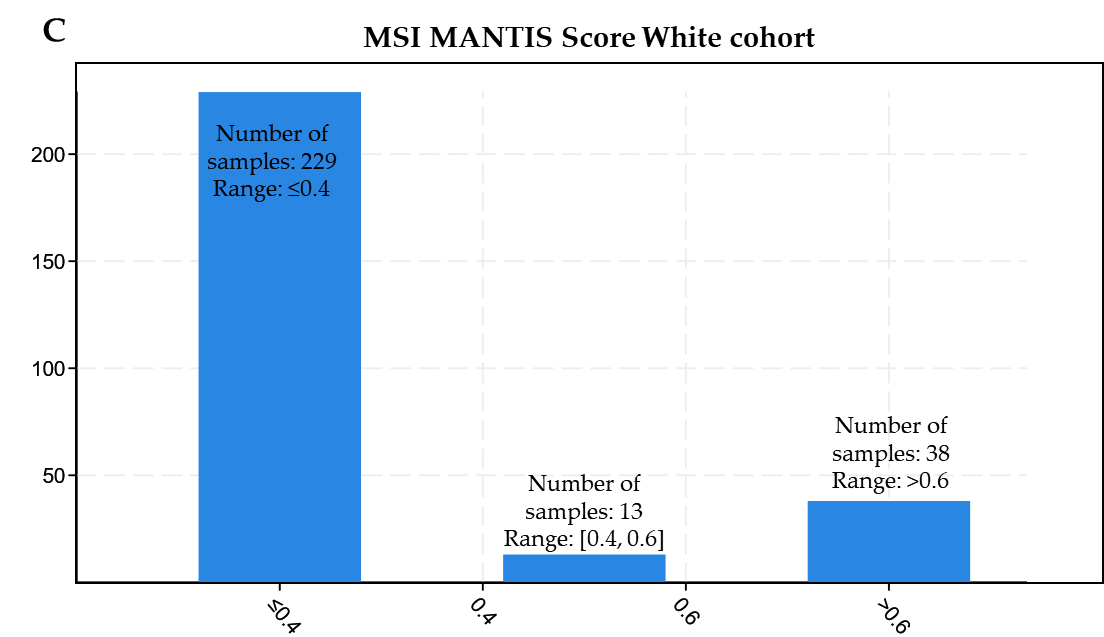
**

**Figure S3. MSI MANTIS Score distribution for each patient cohort Black/AA and White.** MSI MANTIS score distributions for **(A)** both cohorts **(B)** the Black/AA cohort and **(C)** the White cohort. The suggested thresholds are MSI: >0.6, Indeterminate: 0.4 - 0.6 and MSS: <0.4.
