# Supplementary material for: A Systems Biology Approach to Understand the Racial Disparities in Colorectal Cancer
Source: Cancer Res Commun. 2024 Jan 12;4(1):103–17. doi: 10.1158/2767-9764.CRC-22-0464 (PMC10785768; doi:10.1158/2767-9764.CRC-22-0464)
Supplement: Supplementary Figure S4 — shows the probability of overall survival for the MSI MANTIS Scores for each patient cohort [file crc-22-0464-s12.docx]

Supplementary Figure S4

***
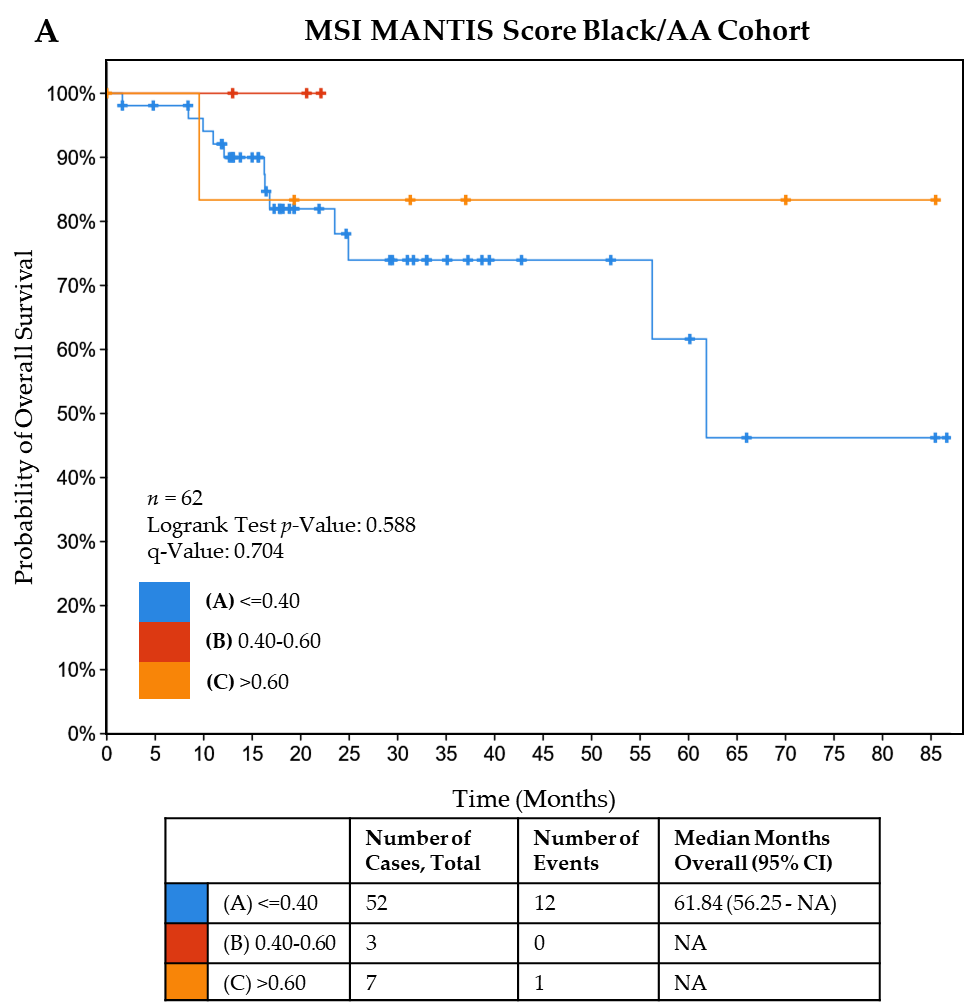

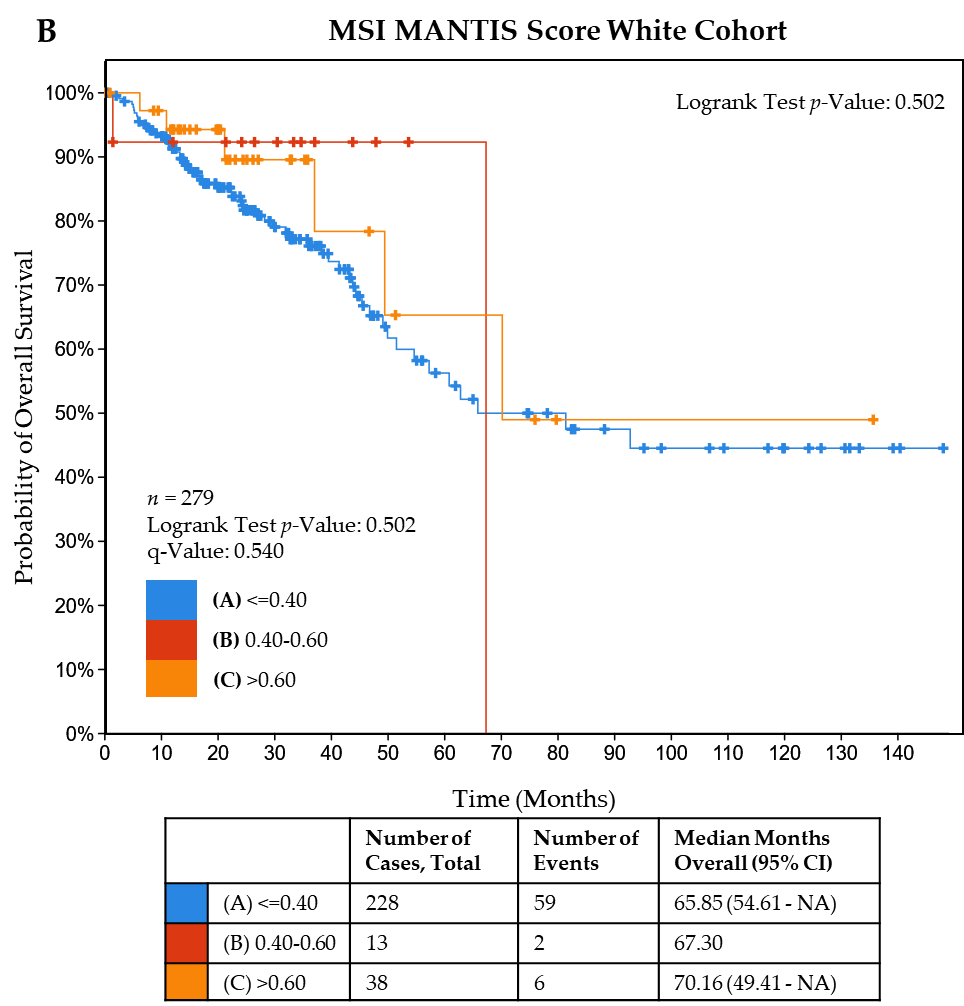
***

**Figure S4. The Probability of Overall Survival for the MSI MANTIS Scores for each patient cohort.** Probability of overall survival for the **(A)** Black/AA cohort and **(B)** White cohort. The suggested thresholds are MSI: >0.6, Indeterminate: 0.4 - 0.6 and MSS: <0. *n* indicates the total number of patients in each group. Statistical significance was tested using a log-rank test. $\mathrm{CI}_{95\%}$= 95% confidence interval.
