# Supplementary material for: A Systems Biology Approach to Understand the Racial Disparities in Colorectal Cancer
Source: Cancer Res Commun. 2024 Jan 12;4(1):103–17. doi: 10.1158/2767-9764.CRC-22-0464 (PMC10785768; doi:10.1158/2767-9764.CRC-22-0464)
Supplement: Supplementary Figure S5 — shows the distribution of the log2 Hazard Ratios from Black/AA and White cohorts [file crc-22-0464-s13.docx]

Supplementary Figure S5

*
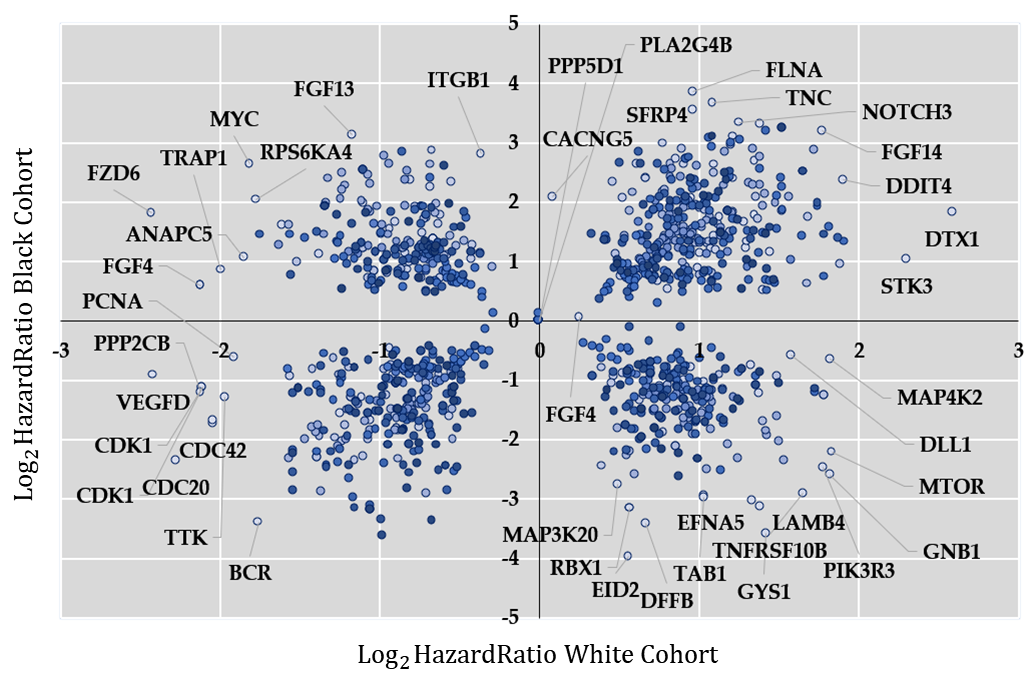
*

**Figure S5. Distribution of the** $\mathbf{log}_{\mathbf{2}}\mathbf{Hazard Ratios}$ **from Black/AA and White cohorts.** The zoomed in region of all the genes analysed in each STN combined (from panel A of main paper: Fig. 1A). Markers vary colour by point.
