# Supplementary material for: A Systems Biology Approach to Understand the Racial Disparities in Colorectal Cancer
Source: Cancer Res Commun. 2024 Jan 12;4(1):103–17. doi: 10.1158/2767-9764.CRC-22-0464 (PMC10785768; doi:10.1158/2767-9764.CRC-22-0464)
Supplement: Supplementary Figure S6 — shows the mRNA distributions for the most significant gene of each CRC STN found in the Black/AA and White patient cohorts [file crc-22-0464-s14.docx]

Supplementary Figure S6


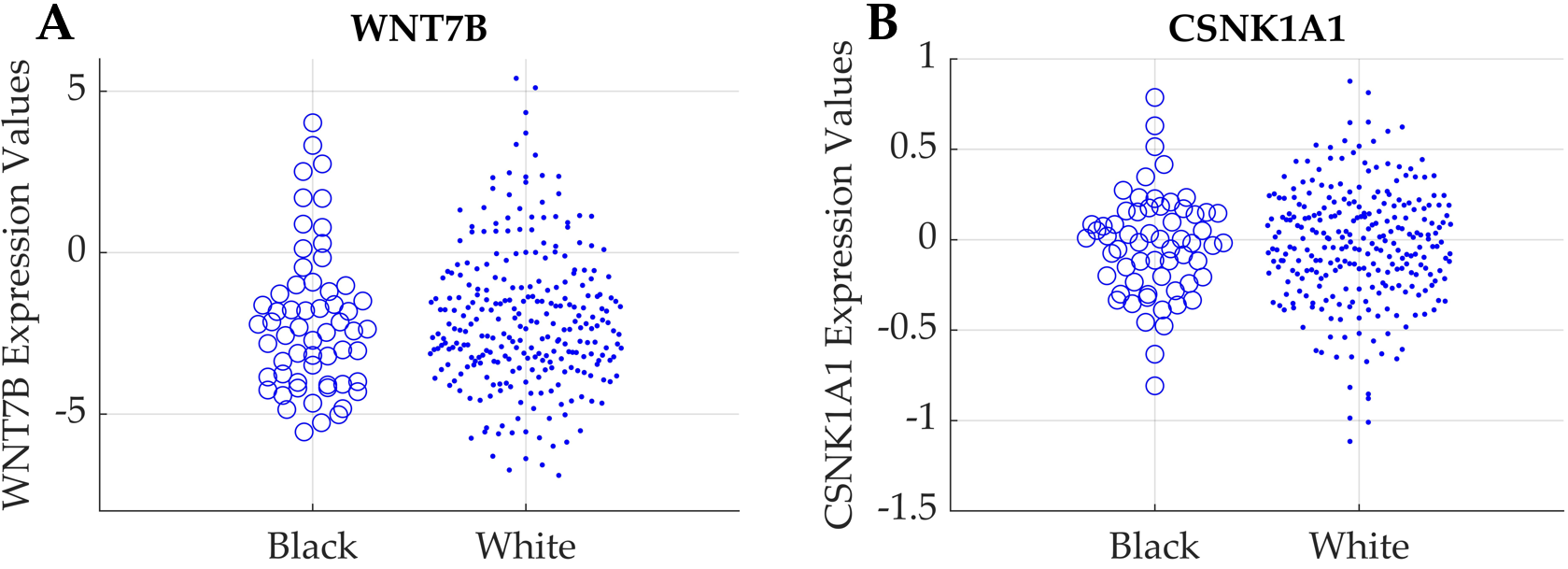


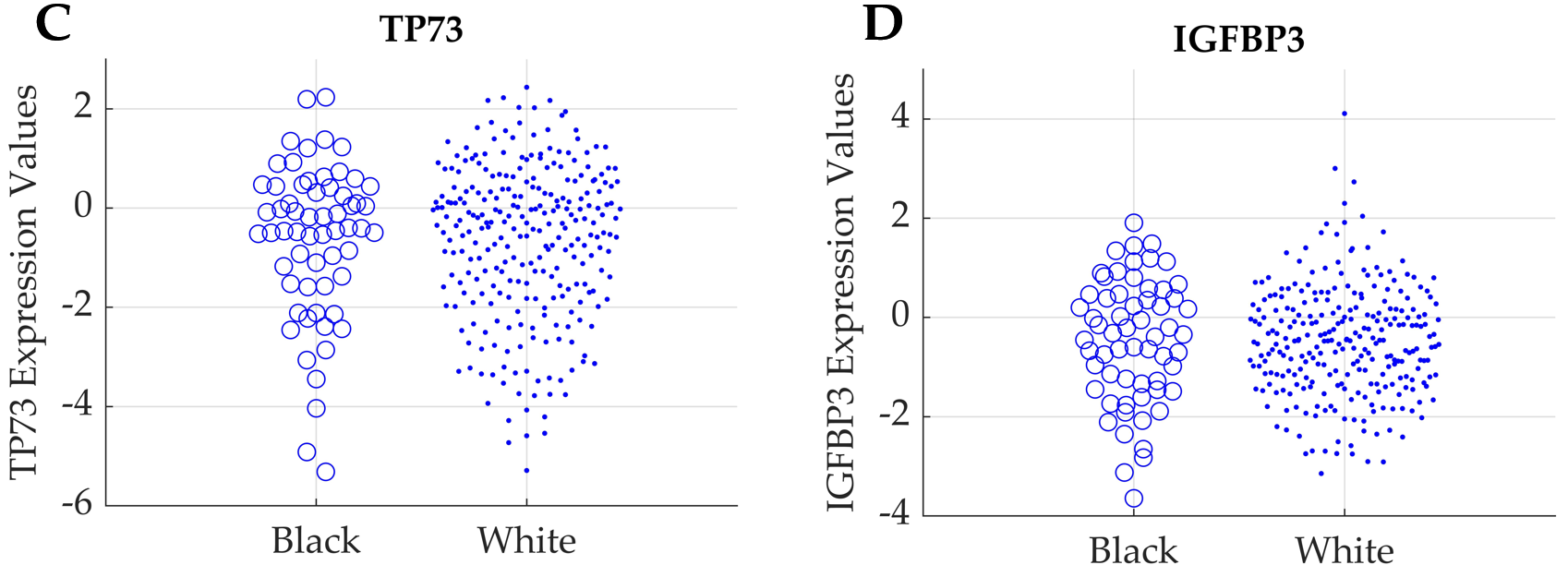


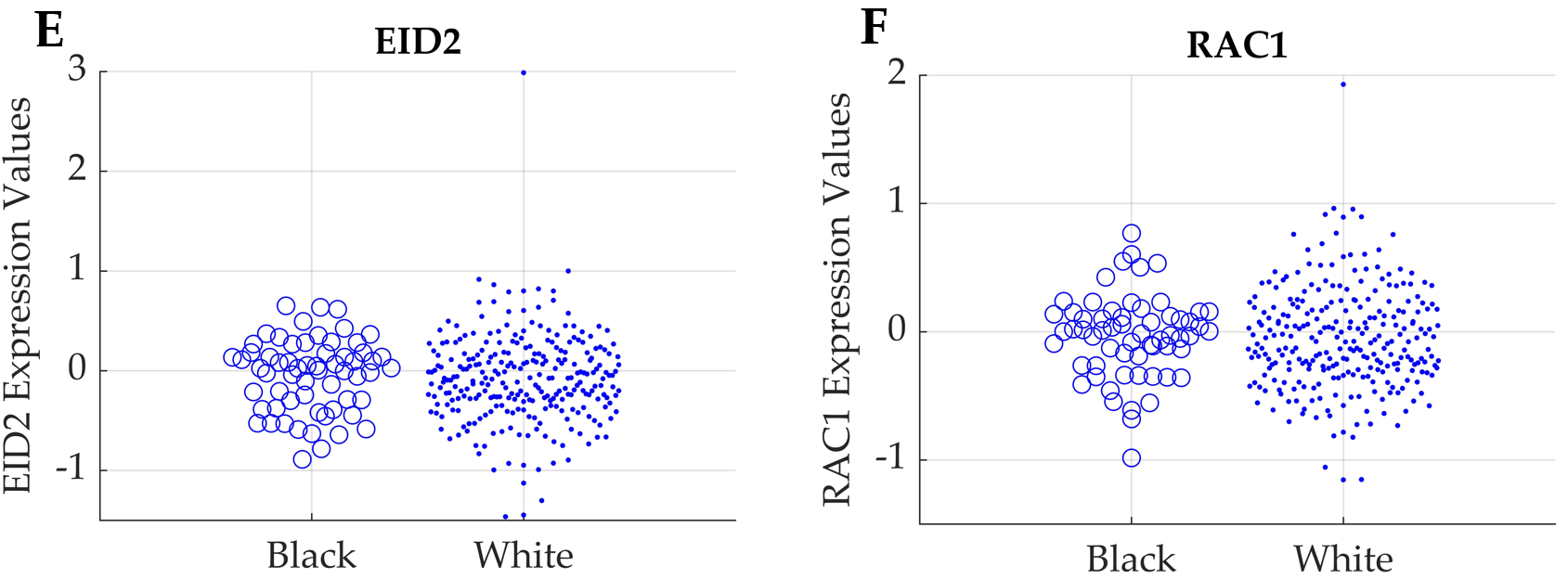


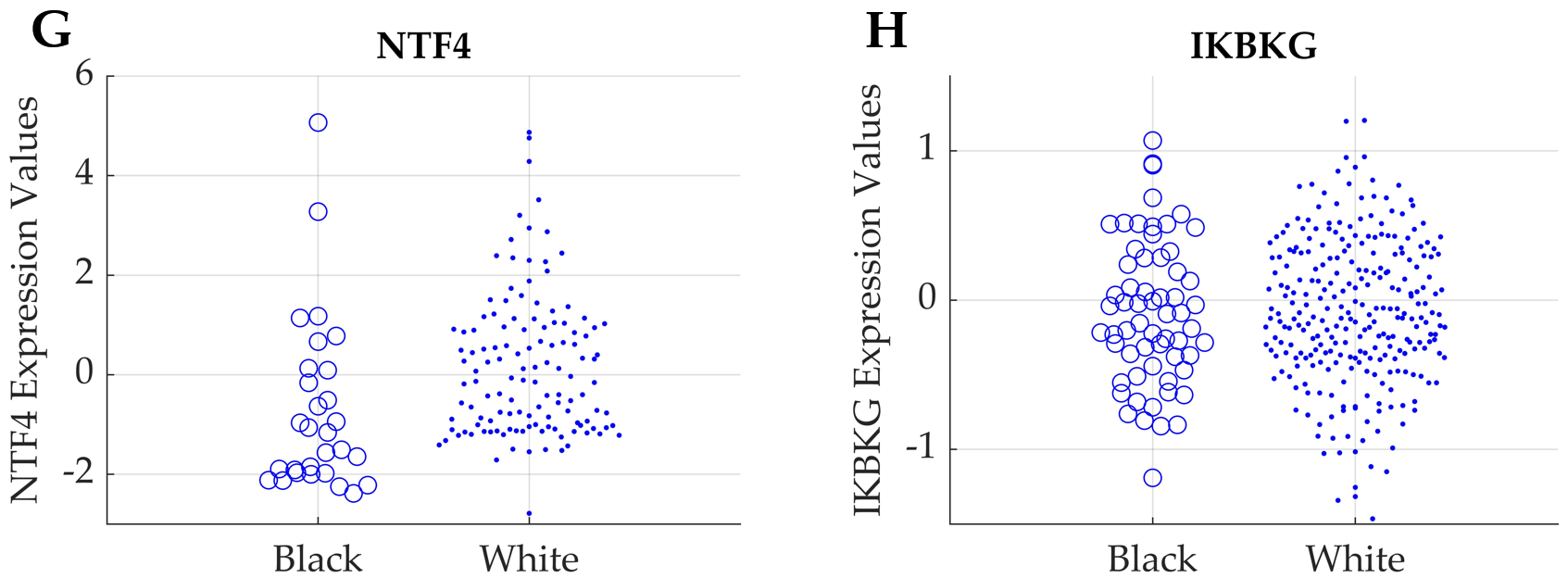


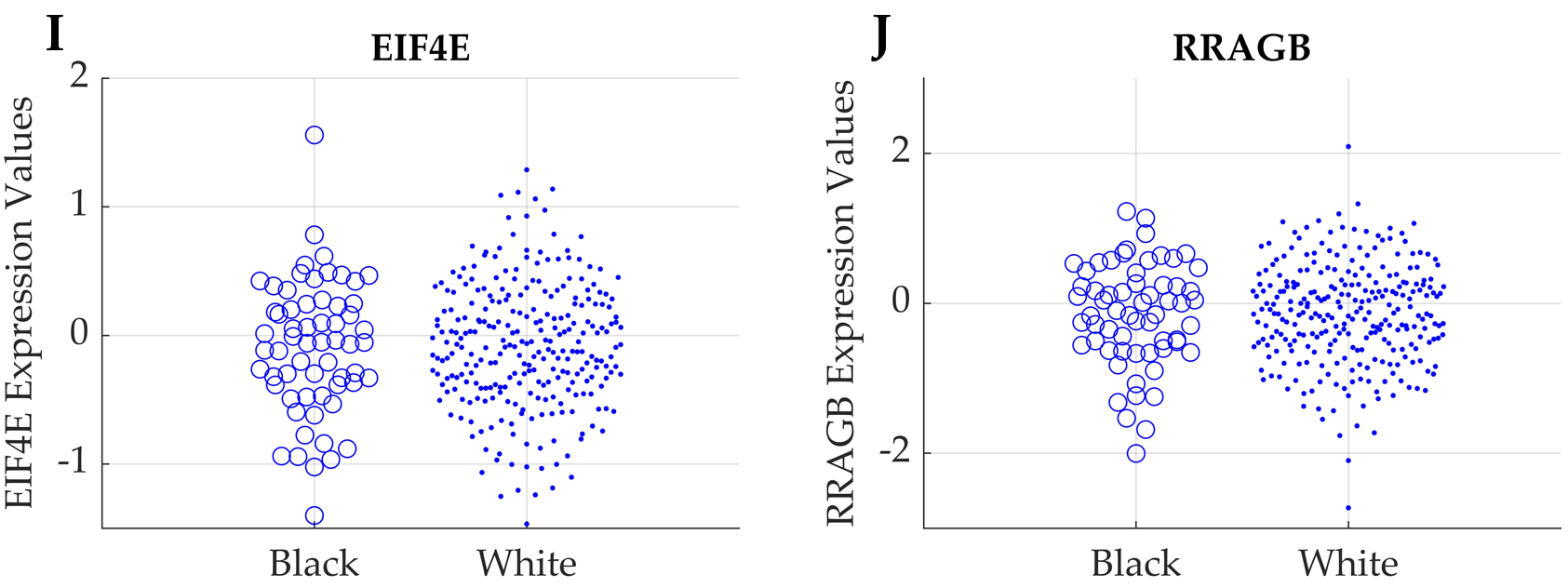


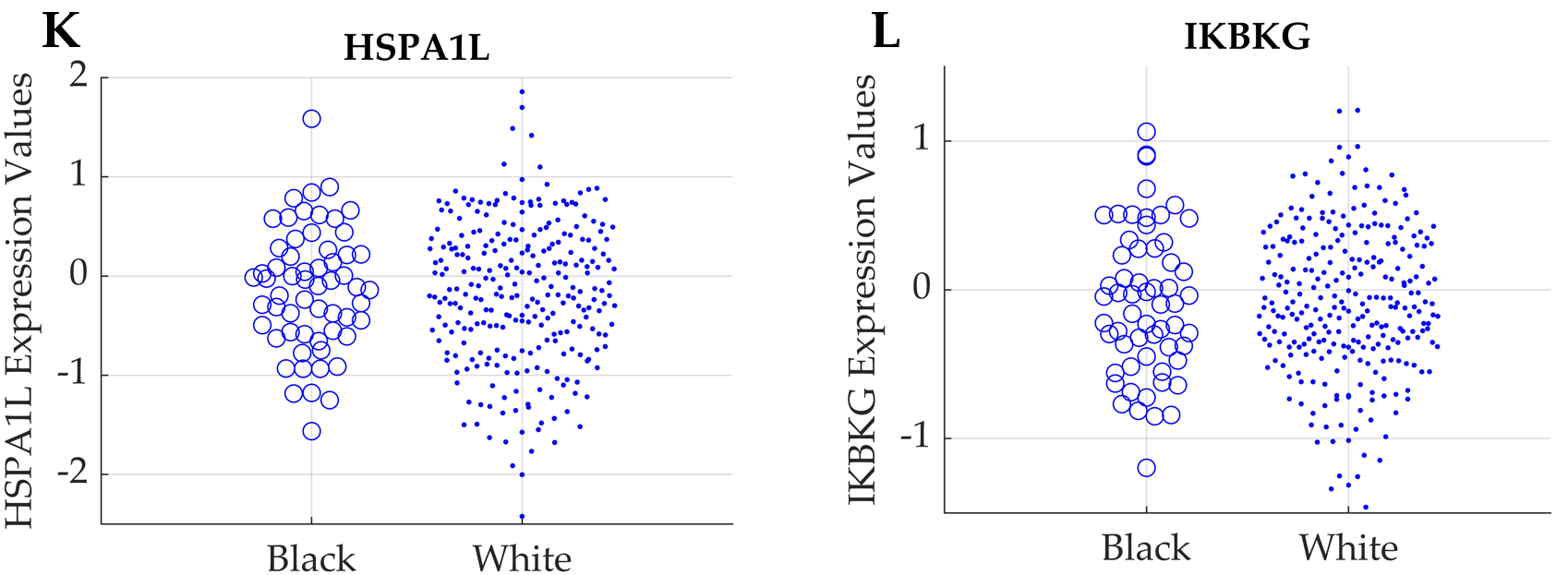


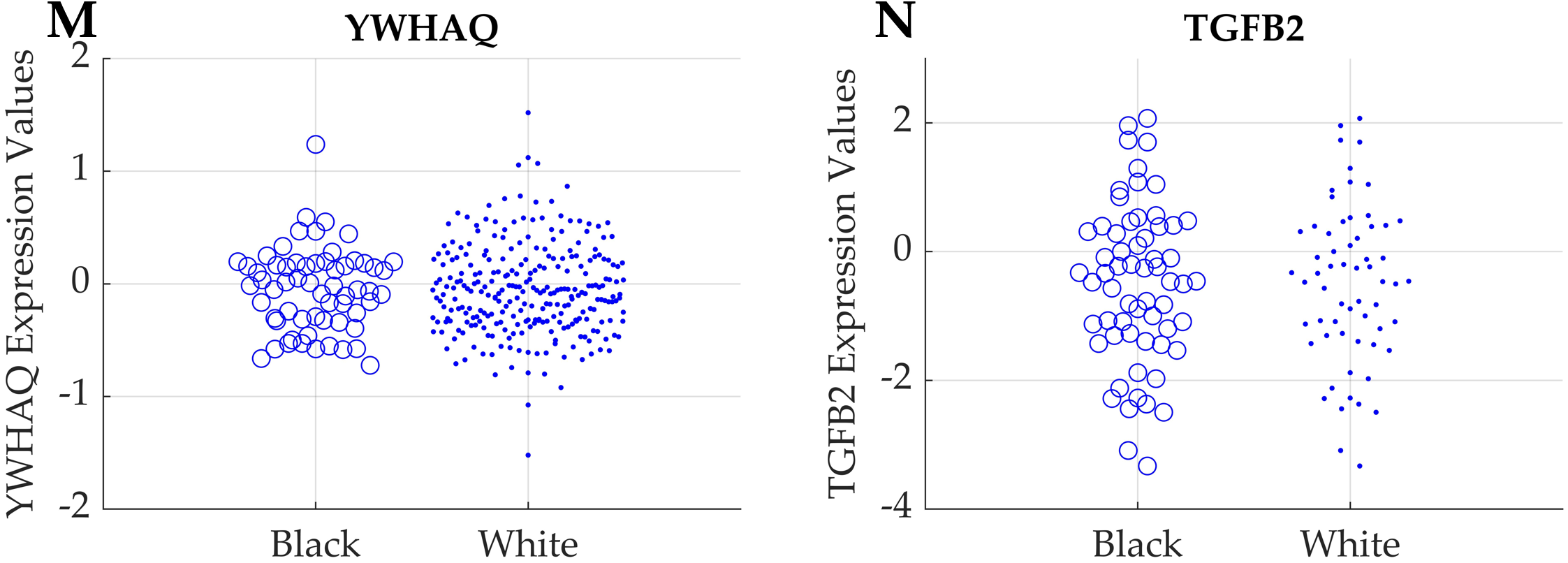


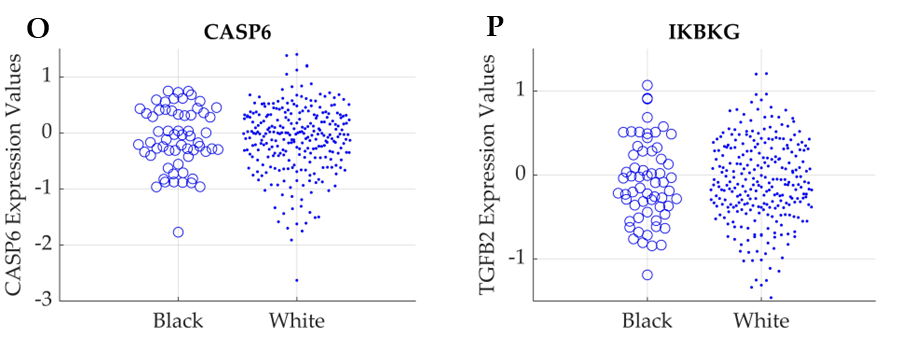


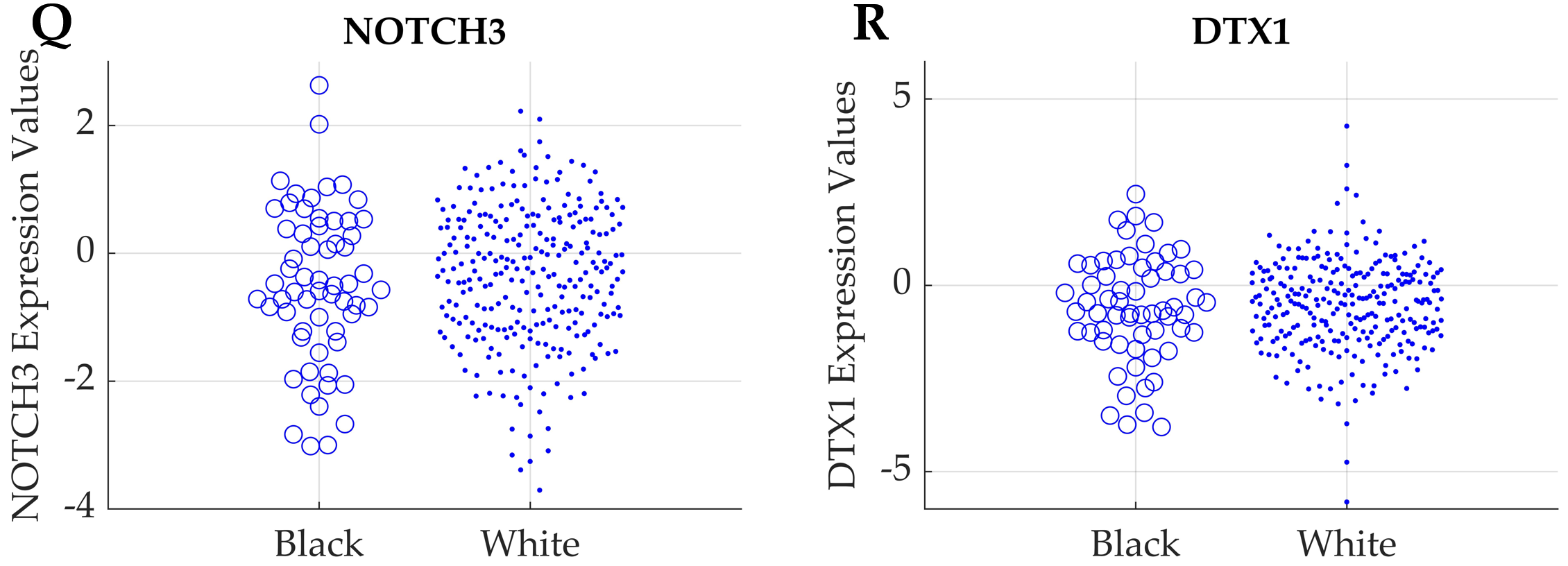


**Figure S6. mRNA distributions for the most significant gene of each CRC STN found in the Black/AA and White patient cohorts**. Distributions for the Black/AA cohort include, **(A)** WNT7B for WNT STN, **(C)** TP73 for TP53 STN, **(E)** EID2 for TGF-Beta STN, **(G)** NTF4 for PI3K-Akt STN, **(I)** EIF4E for mTOR STN, **(K)** HSPA1L for MAPK STN, **(M)** YWHAQ for Cell Cycle STN, **(O)** CASP6 for Apoptosis STN, and **(Q)** NOTCH3 for Notch STN. Distributions for the White cohort include, **(B)** CSNK1A1 for WNT STN, **(D)** IGFBP3 for TP53 STN, **(F)** RAC1 for TGF-Beta STN, **(H)** IKBKG for PI3K-Akt STN, **(J)** RRAGB for mTOR STN, **(L)** IKBKG for MAPK STN, **(N)** TGFB2 for Cell Cycle STN, **(P)** IKBKG for Apoptosis STN, and **(R)** DTX1 for Notch STN. The first (left most distribution) is the most significant gene distribution for the Black/AA cohort and the second (right most distribution) is for the equivalent for the White cohort. The y-axis represents the $\log_{2}$of the gene expression values. The x-axis indicates the representing cohort.
