# Supplementary material for: A Systems Biology Approach to Understand the Racial Disparities in Colorectal Cancer
Source: Cancer Res Commun. 2024 Jan 12;4(1):103–17. doi: 10.1158/2767-9764.CRC-22-0464 (PMC10785768; doi:10.1158/2767-9764.CRC-22-0464)
Supplement: Supplementary Figure S7 — shows a heatmap of the differentially expressed genes between the Black/AA and White cohorts [file crc-22-0464-s15.docx]

Supplementary Figure S7


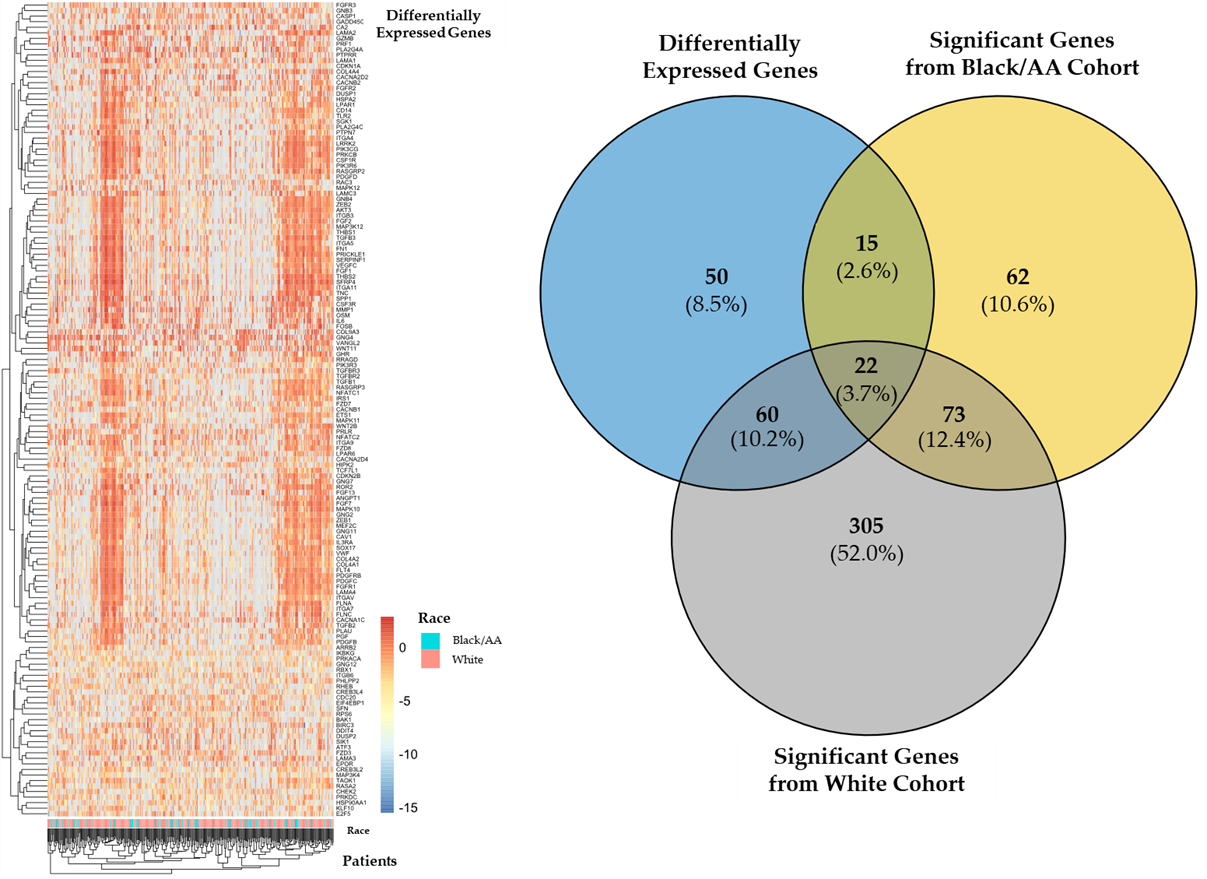


**Figure S7. Heatmap of the differentially expressed genes between the Black/AA and White cohorts.** The y-axis and x-axis represent the differentially expressed genes and the associated patients, respectively. Euclidean distance and complete linkage were used to create the hierarchical cluster tree.
