# Supplementary material for: A Systems Biology Approach to Understand the Racial Disparities in Colorectal Cancer
Source: Cancer Res Commun. 2024 Jan 12;4(1):103–17. doi: 10.1158/2767-9764.CRC-22-0464 (PMC10785768; doi:10.1158/2767-9764.CRC-22-0464)
Supplement: Supplementary Figure S8 — shows the PROGENy pathway activity scores from the mRNA expressions for Black/AA and White patient cohorts [file crc-22-0464-s16.docx]

Supplementary Figure S8


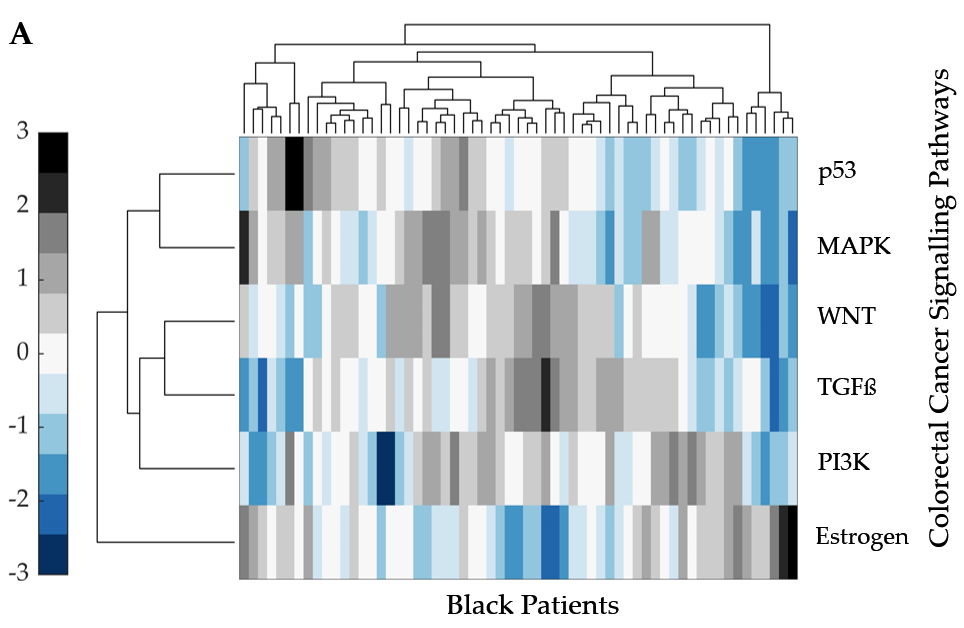


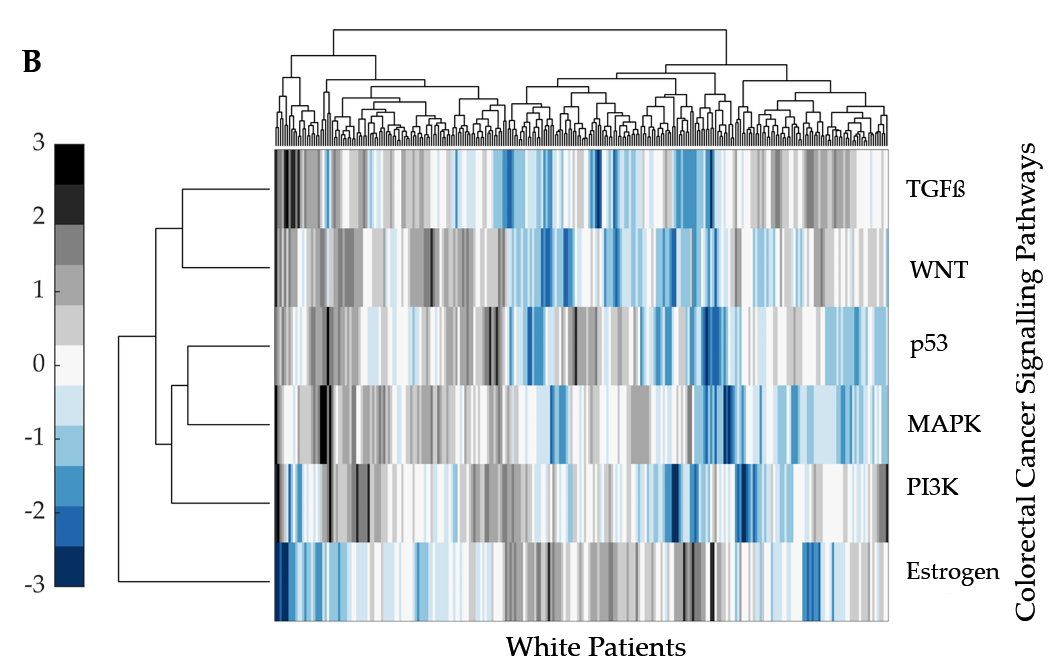


**Figure S8. PROGENy pathway activity scores from the mRNA expressions for Black/AA and White patient cohorts.** Heatmaps of the pathway activity scores (*z*-coefficients) for all **(A)** Black/AA patients and **(B)** White patients**.** Euclidean distance and complete linkage were used to create the hierarchical cluster tree.
