# Supplementary material for: A Systems Biology Approach to Understand the Racial Disparities in Colorectal Cancer
Source: Cancer Res Commun. 2024 Jan 12;4(1):103–17. doi: 10.1158/2767-9764.CRC-22-0464 (PMC10785768; doi:10.1158/2767-9764.CRC-22-0464)
Supplement: Supplementary Figure S9 — shows the overview of the effects of WNT pathway activity scores and APC alternative event frequency between Black/AA and White patient cohorts [file crc-22-0464-s17.docx]

Supplementary Figure S9


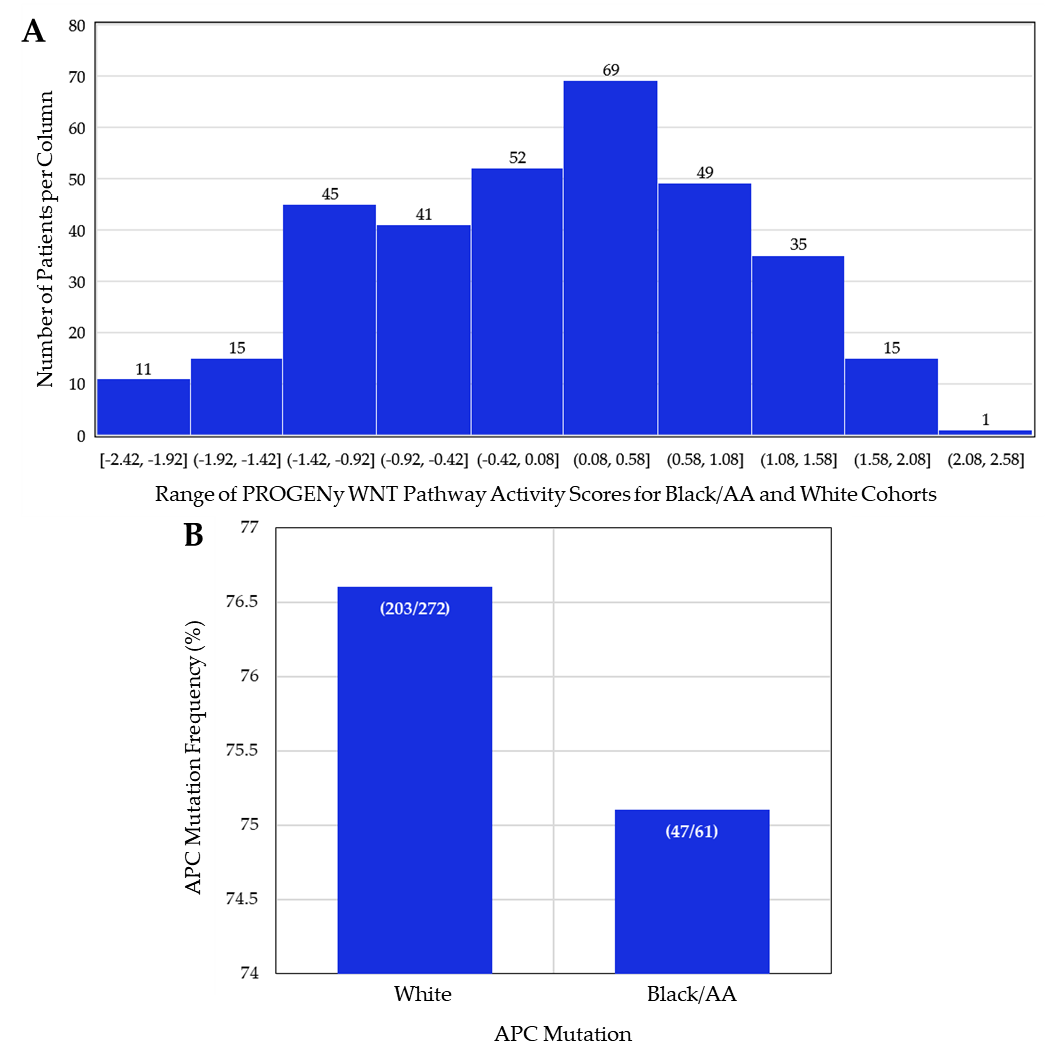


**Figure S9. Overview of the effects of WNT pathway activity scores and APC alternative event frequency between Black/AA and White patient cohorts. (A)** The range of PROGENy WNT pathway activity scores within both cohorts and **(B)** The frequency of APC mutations within both cohorts (Black/AA = 76.5% and White = 75.1%). *p*-Value =0.472. q-Value = 0.754
